# Cytotoxic Pentaketide-Sesquiterpenes from the Marine-Derived Fungus *Talaromyces variabilis* M22734

**DOI:** 10.3390/md22060274

**Published:** 2024-06-13

**Authors:** Lingzhi Tang, Jinmei Xia, Zhongwei Chen, Xiaohui Wu, Guangyu Li, Qiliang Lai, Zongze Shao, Weiyi Wang, Xuan Hong

**Affiliations:** 1 Xiamen Key Laboratory of Marine Medicinal Natural Products Resources, Xiamen Medical College, Xiamen 361023, China; tlz@xmmc.edu.cn (L.T.); czw@xmmc.edu.cn (Z.C.); wxh18760630472@163.com (X.W.); 2 Key Laboratory of Marine Biogenetic Resources, Third Institute of Oceanography, Ministry of Natural Resources, Xiamen 361005, China; xiajinmei@tio.org.cn (J.X.); liguangyu@tio.org.cn (G.L.); laiqiliang@tio.org.cn (Q.L.); shaozongze@tio.org.cn (Z.S.); 3 School of Pharmacy, Fujian Medical University, Fuzhou 350122, China

**Keywords:** talaromyces, pentaketide-sesquiterpenes, cytotoxic, marine

## Abstract

*Talaromyces*, a filamentous fungus widely distributed across terrestrial and marine environments, can produce a diverse array of natural products, including alkaloids, polyketones, and polyketide-terpenoids. Among these, chrodrimanins represented a typical class of natural products. In this study, we isolated three previously undescribed pentaketide-sesquiterpenes, 8,9-*epi*-chrodrimanins (**1**–**3**), along with eight known compounds (**4**–**11**). The structures of compounds **1**–**3** were elucidated using nuclear magnetic resonance (NMR) and mass spectrometry (MS), while their absolute configurations were determined through X-ray crystallography and electronic circular dichroism (ECD) computations. The biosynthetic pathways of compounds **1**–**3** initiate with 6-hydroxymellein and involve multiple stages of isoprenylation, cyclization, oxidation, and acetylation. We selected four strains of gastrointestinal cancer cells for activity evaluation. We found that compound **3** selectively inhibited MKN-45, whereas compounds **1** and **2** exhibited no significant inhibitory activity against the four cell lines. These findings suggested that 8,9-*epi*-chrodrimanins could serve as scaffold compounds for further structural modifications, potentially leading to the development of targeted therapies for gastric cancer.

## 1. Introduction

The genus *Talaromyces*, a member of the *Trichocomaceae* family, was first identified in 1955 by the American mycologist Chester Ray Benjamin. Species within this genus produce ascocarps characterized by soft, cotton-like structures with densely woven hyphal walls. These ascocarps often display a yellowish tint or are surrounded by similarly colored granules [1]. Among the secondary metabolites produced by *Talaromyces* are alkaloids, peptides, lactones, polyketides, and polyketide-terpenoids. Polyketide-terpenoids, a unique class of natural products, feature a biosynthetic pathway that combines polyketides and terpenes, exhibiting a broad range of biological activities [2]. In an investigation involving the *Talaromyces* sp. YO-2 strain, researchers discovered a novel pentaketide-drimane named chrodrimanin C and reisolated the known pentaketide-drimanes, chrodrimanins A and B. Among these, chrodrimanin B demonstrated significant insecticidal properties with an LD_50_ of 10 μg/g in dietary tests, while chrodrimanins A and C showed no activity. Subsequent studies uncovered four additional pentaketide-drimanes, designated as chrodrimanins D to G, together with the previously identified chrodrimanin H. Importantly, chrodrimanins D, E, and F exhibited insecticidal effectiveness against silkworms, with LD_50_ values of 20, 10, and 50 μg/g of diet, respectively [3,4,5].

In recent years, obtaining microbial resources from unique habitats to develop natural drugs has been a hot trend in drug development. The distinct environmental factors of the marine environment, such as high pressure, low temperature, the absence of light, and partially oligotrophic conditions, have shaped unique metabolic mechanisms in microbes. These mechanisms are crucial for the production of natural products with new structures, meaning that marine-derived microbes are considered significant sources of these novel natural products [6,7].

**Figure 1:**
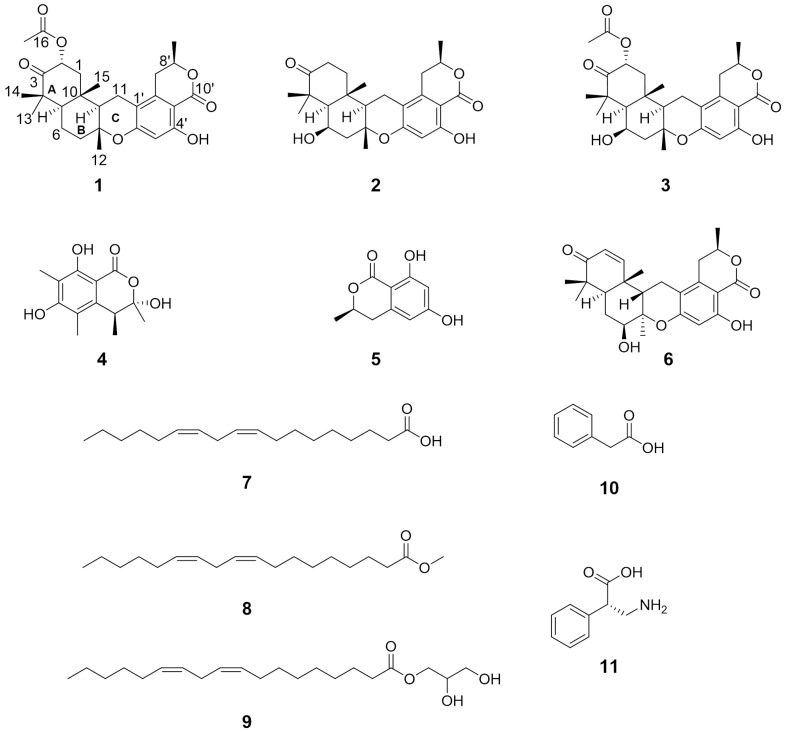
The structures of compounds **1**–**11**.

In our exploration of bioactive compounds originating from the marine environment, we isolated three previously undescribed 8,9-*epi*-chrodrimanin derivatives (Figure 1) from a marine-derived strain of *Talaromyces variabilis* M22734. Their absolute configurations were determined through spectral analysis, X-ray crystallography, and ECD calculation. Furthermore, their biosynthetic pathways were deduced, and their cytotoxic activities were preliminarily evaluated.

## 2. Results

**Figure 2:**
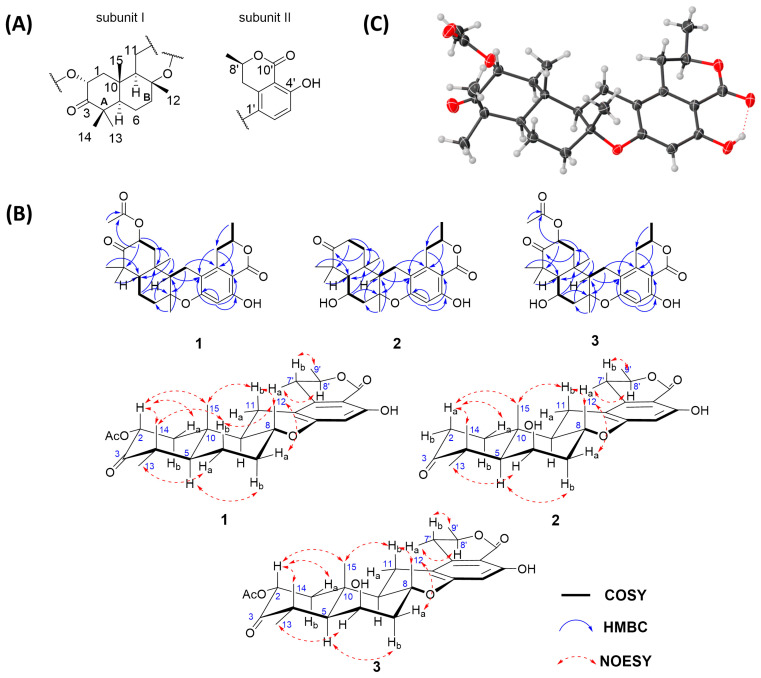
(**A**) Two subunits of **1**; (**B**) key HMBC, COSY, and NOESY correlations of **1**–**3**; (**C**) X-ray structure of **1**.

Compound **1** was characterized by its proton adduct ion (*m/z* 471.2369, Figure S1), from which the molecular formula C_27_H_34_O_7_ was deduced. This formula suggested eleven degrees of unsaturation. Extensive spectroscopic analysis including ^1^H and ^13^C NMR, HSQC, and ^13^C/DEPT (Figures S2−S5) revealed distinct structural elements (Table 1): one ketone carbonyl (*δ*_C_ 208.0), three quaternary sp^3^ carbons with one oxygenated (*δ*_C_ 48.5, 77.2, 37.5), two ester carbonyls (*δ*_C_ 170.2, 170.0), five aromatic sp^2^ carbons (*δ*_C_ 101.9, 139.1, 110.1, 162.4, 160.1), four sp^3^ methines with two oxygenated (*δ*_C/H_ 71.1/5.68, 56.6/1.47, 51.2/1.70, 74.6/4.64), one aromatic sp^2^ methine (*δ*_C/H_ 103.5/6.29), and five endocyclic methylenes (*δ*_C/H_ 44.2/2.38 and 1.57, 19.9/1.78 and 1.63, 40.1/2.16 and 1.72, 19.6/2.53 and 2.35, 31.4/2.93 and 2.63), along with six methyl groups (*δ*_C/H_ 21.0/1.25, 25.0/1.18, 21.2/1.19, 15.3/1.30, 20.8/2.17, 20.9/1.55). COSY (Figure S7) correlation analysis revealed four spin systems: C-1/C-2, C-5/C-6/C-7, C-9/C-11, and C-7′/C-8′/C-9′, as depicted in Figure 2B. HMBC (Figure S6) correlations allowed for the assignment of subunit I from correlations such as H-2 to C-4/C-10, H_3_-13(14) to C-5, H_2_-6 to C-8/C-10, H_2_-7 to C-9, H_3_-12 to C-7/C-9, and H_3_-15 to C-1/C-5/C-9 (Figure 2A). Partial subunit II was resolved through HMBC correlations of H_3_-9′ to C-7′, H-8′ to C-2′, H_2_-7′ to C-1′/C-3′, and H-5′ to C-1′/C-3′/C-4′/C-6′ (Figure 2A). From the chemical shift *δ*_H_ 11.11, it was inferred that a hydroxyl group formed a hydrogen bond with a carbonyl group, causing a downfield shift. This indicated the presence of one ester carbonyl in subunit II at a meta position relative to OH-4′ (Table 1). Another ester carbonyl was contributed by the acetyl group attached to C-2, as derived from the HMBC correlation of H-2 with C-16 (Figure 2B). HMBC correlations of H-9 with C-1′ and H_2_-11 to C-2′ (Figure 2B) indicated the linkage of subunits I and II to C-11-C-1′. Based on the structural data available, the combination of subunits I and II resulted in a total molecular composition of 27 carbons, 34 hydrogens, and 6 oxygens. Subunit I exhibited an unsaturation degree of four, while subunit II displayed a degree of six. Given the molecular formula C_27_H_34_O_7_, there likely existed an additional oxygen bridge that contributed to the final degree of unsaturation. Analysis of the downfield chemical shifts at C-8 and C-6′ led to the deduction that these carbons were connected via an oxygen bridge. This connection, along with C-9, C-11, and C-1′, formed an oxygen-containing six-membered ring that linked subunits I and II.

NOESY (Figure S8) correlations (e.g., H_3_-15/H-2, H-2/H_3_-14, H_3_-14/H_b_-6, H_b_-6/H_3_-12, H_a_-7/H_3_-12, H_3_-12/H_b_-11, and H_b_-11/H_3_-15) indicated these protons were oriented on the same face of the ring system, tentatively assigned as *β*. Conversely, H_3_-13/H_a_-6 and H-5/H_b_-7 indicated an *α* orientation for these protons (Figure 2B). The relative stereochemistry of the isolated chiral center C-8′ could not be determined using NOESY signals alone; fortunately, the absolute configuration was established via X-ray crystallography (Figure 2C) with Cu Kα radiation (Flack parameter = 0.00(5); CCDC number: 2354719). Thus, compound **1** was confirmed as (2*R*,5*R*,8*R*,9*R*,10*S*,8′*R*)-2-acetyl-chrodrimanin C, belonging to the 8,9-*epi*-chrodrimanin derivatives.

**Figure 3:**
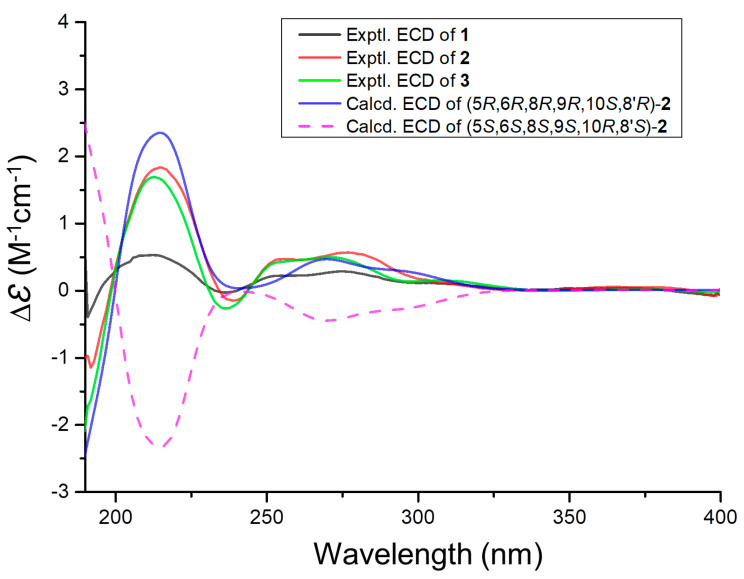
Experimental ECD spectra of **1**–**3** and calculated ECD spectra of **2**.

Compound **2** was characterized by a proton adduct ion, *m/z* 429.2280 (Figure S9), from which the molecular formula C_25_H_32_O_6_ was inferred, indicating a total of ten degrees of unsaturation. Through careful comparison of the NMR data of compounds **2** (Figures S10−S13) and **1**, it was observed that compound **2** lacked the signal for one acetyl group and showed differences at C-1, C-2, C-3, C-6, C-7, and C-14, suggesting that the primary structural differences between compounds **2** and **1** reside in rings A and B (Table 1). The 2D NMR data of compounds **2** and **1** were very similar; notably, COSY (Figure S15) correlations of H-5/H-6/H_2_-7 and HMBC (Figure S14) correlations of H-6 with C-8/C-10 allowed the deduction of an OH group attached to C-6 (*δ*_C/H_ 68.4/4.61) (Figure 2B). ^3^*J*_HH_ values for H_b_-11/H-9 and H_b_-7′/H-8′ were 13.1 and 11.5 Hz, respectively, indicating the axial orientation for H-11b and H-7′b, and tentatively assigned a *β* orientation (Table 1). The NOESY (Figure S16) correlations of H_3_-15/H_a_-2, H_a_-2/H_3_-14, H_a_-7/H_3_-12, H_3_-12/H_b_-11, H_b_-11/H_3_-15, and H_3_-9′/H_b_-7′ indicated that these protons were oriented on the same face of the ring system, tentatively assigned a *β* orientation, whereas H_3_-13/H-6 and H-5/H_b_-7 indicated an orientation on the opposite face, tentatively assigned an α orientation (Figure 2B). Subsequently, the conformer 5*R*,6*R*,8*R*,9*R*,10*S*,8′*R*-**2** and its epimer underwent ECD calculations at the CAM-B3LYP/6-311G(d) level (Tables S1−S13). It was found that the experimental results corresponded more closely with the structure of 5*R*,6*R*,8*R*,9*R*,10*S*,8′*R*-**2**. Consequently, the absolute configuration was determined to be 5*R*,6*R*,8*R*,9*R*,10*S*,8′*R*. (Figure 3).

Compound **3** was characterized by a sodium adduct ion, *m/z* 509.2156 (Figure S17), from which the molecular formula C_27_H_34_O_8_ was inferred, indicating a total of eleven degrees of unsaturation. Through careful comparison of the NMR data between compounds **3** (Figures S18−S21) and **2**, it was observed that compound **3** displayed an additional signal for an acetyl group, while other chemical shifts were very close. The 2D NMR data for compounds **3** and **2** were also very similar; however, notable distinctions included the COSY (Figure S23) correlations of H_2_-1 with H-2 and the HMBC (Figure S22) correlations of H-2 with C-4, C-10, and C-16, which elucidated that C-2 (*δ*_C/H_ 71.2/5.79) was linked to an acetyl group (Figure 2B). The NOESY (Figure S24) correlations involving H_3_-15 with H-2, H-2 with H_3_-14, H_a_-7 with H_3_-12, H_3_-12 with H_b_-11, H_b_-11 with H_3_-15, and H_3_-9′ with H_b_-7′ indicated that these protons were oriented on the same face of the ring system. In contrast, the correlations between H_3_-13 and H-6 and H-5 and H_b_-7 showed that these protons were oriented on the opposite face, similar to compound **1** (Figure 2B). The orientation of the OH group connected to C-6 was consistent with that in compound **2**. From the highly consistent ECD spectra of compounds **1** to **3**, it was determined that the absolute configuration of compound **3** at the corresponding positions was consistent with compounds **1** and **2** as 2*R*,5*R*,6*R*,8R,9*R*,10*S*,8′*R* (Figure 3).

Comparative analysis of NMR data with the reported literature identified compounds **4**–**11** as (3*S*,4*S*)-sclerotinin A (**4**) [8], (*R*)-6-hydroxymellein (**5**) [9], chrodrimanin E (**6**) [3], (9*Z*,12*Z*)-octadeca-9,12-dienoic acid (**7**) [10], methyl (9*Z*,12*Z*)-octadeca-9,12-dienoate (**8**) [11], 2,3-dihydroxypropyl (9*Z*,12*Z*)-octadecadienoate (**9**) [12], phenyl acetic acid (**10**) [13], and (*S*)-3-amino-2-phenylpropionic acid (**11**) [14], respectively.

**Figure 4:**
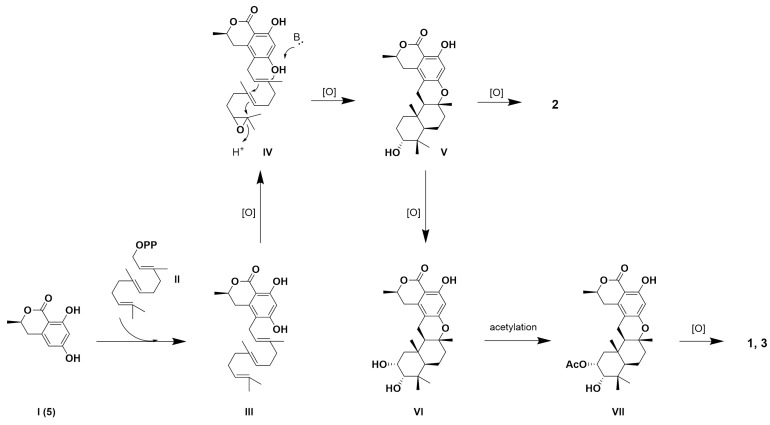
Biosynthetic pathways of **1**–**3**.

The biosynthetic pathways for compounds **1**–**3** were proposed to have originated from 6-hydroxymellein (**5**, **I**). Through isoprenylation, intermediate **III** was formed, followed by cyclization and oxidation to yield intermediate **V**. A part of intermediate **V** underwent further oxidation to produce compound **2**, while another portion underwent additional oxidation and acetylation, proceeding through intermediates **VI** and **VII**, to yield compounds **1** and **3** (Figure 4).

**Figure 5:**
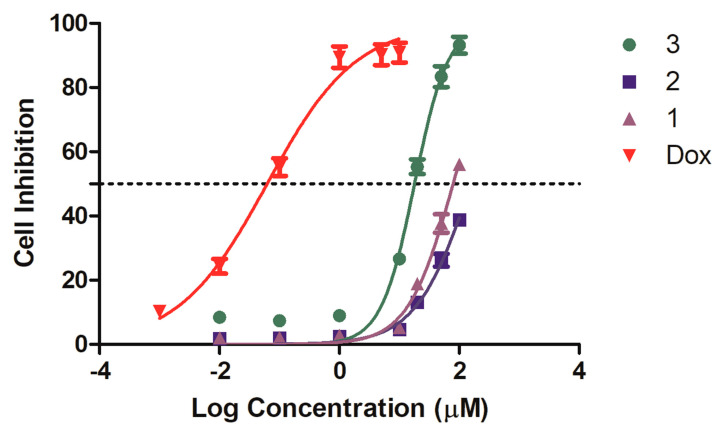
Compound **3** inhibited the proliferation of MKN-45 cells in the CCK8 assay.

At 20 μM, compound **3** exhibited selective inhibitory activity against four strains of gastrointestinal cancer cells, achieving a 55.3% inhibition rate in MKN-45 cells. In contrast, the positive control drug, doxorubicin (Dox), demonstrated a non-selective inhibition rate exceeding 75% for all tested strains (Table 2). Compounds **1** and **2** displayed significantly lower inhibition rates, below 20% for all strains. Subsequent IC_50_ determinations for compounds **1**–**3** and Dox on MKN-45 cells revealed that compound **3** had an IC_50_ of 18.0 μM, whereas compound **1** had an IC_50_ of 79.2 μM, and compound **2** did not achieve a 50% inhibition rate even at 100 μM. The IC_50_ for Dox was 62.5 nM (Figure 5).

## 3. Discussion

We isolated three previously undescribed 8,9-*epi*-chrodrimanins and established the orientation of two magnetically inequivalent hydrogen atoms on C-11 and C-7′ by analyzing coupling constants and further determined the relative configurations of rings A and B, as well as C-8′, through NOESY. For compound **1**, its absolute configuration was resolved using X-ray crystallography. For compound **2**, the absolute configuration was determined via NMR elucidation combined with ECD calculations. Regarding compound **3**, we first compared its NMR data with those of compounds **1** and **2** to identify structural similarities and differences, and we then determined its relative configuration with the aid of NOESY. The consistency between the ECD spectra of compound **3** and those of compounds **1** and **2** further confirmed its absolute configuration. Furthermore, we assessed the cytotoxic activities of compounds **1**–**3** against four gastrointestinal cancer cell lines: MKN-45, HCT 116, TE-1, and PATU8988T. Compound **3** exhibited selective inhibitory activity against MKN-45 cells, in contrast to the non-selective inhibitory activity of the positive control drug. Compounds **1** and **2** demonstrated weaker inhibitory effects across all tested cell lines.

## 4. Materials and Methods

### 4.1. General Experimental Procedures

NMR data were measured on a Bruker Avance 600 MHz spectrometer (Billerica, MA, USA). HRESIMS were determined using a Xevo G2 Q-TOF mass spectrometer (Waters, Milford, MA, USA) in ESI mode. ECD spectra were recorded using a J-715 spectropolarimeter (JASCO Corporation, Tokyo, Japan). The optical rotations were determined via an Anton Paar MCP 500 polarimeter (Graz, Austria).

### 4.2. Fungal Material

The identity of the strain was confirmed through ITS sequencing, revealing that this strain has a 100% similarity to the *Talaromyces variabilis* strain ZHKUCC 23-1032 (Genbank accession No. PP029409.1). The original designation of the strain was R3-HSHG1, and after deposition, it was cataloged under the preservation number M22734.

### 4.3. Fermentation, Extraction, and Isolation

Solid-state fermentation was conducted on the strain *Talaromyces variabilis* M22734 over 25 days. A total of 120 one-liter Erlenmeyer flasks were utilized, each containing 100 g of rice and 100 milliliters of seawater, maintained at 28 °C. Subsequently, three rounds of extraction were performed on each flask using 200 milliliters of ethyl acetate. The extracts were concentrated under vacuum to dryness, yielding approximately 175 g of total extract. This crude extract was subsequently subjected to chromatographic separation on a silica gel column, employing a petroleum ether–ethyl acetate gradient from 9.7:0.3 to 8.0:2.0, producing 15 fractions (Fr1 to Fr15). Fraction Fr5 was further subdivided into Fr5-1 to Fr5-4 via Sephadex LH-20 chromatography in methanol. From these, Fr5-3 was isolated through reverse-phase HPLC using a gradient of 20–80% acetonitrile in water over 20.0 min at a flow rate of 10 mL/min, yielding compound **6** (8.7 mg, *t*_R_ = 16.9 min). Similarly, Fr5-4 yielded compound **5** (4.8 mg, *t*_R_ = 16.3 min) under the same conditions. Fr6 was partitioned into Fr6-1 to Fr6-4, with Fr6-4 yielding compounds **2** (1.4 mg, *t*_R_ = 26.5 min), **1** (4.4 mg, *t*_R_ = 28.2 min), **4** (3.1 mg, *t*_R_ = 19.7 min), and **3** (4.0 mg, *t*_R_ = 21.8 min) through a multi-step HPLC process. Fr7 was separated into Fr7-1 to Fr7-3, with compound **7** (151.5 mg, *t*_R_ = 17.5 min) and compound **8** (18.2 mg, *t*_R_ = 24.9 min) isolated from Fr7-3. Fr12 was subdivided into Fr12-1 to Fr12-4, with Fr12-2 yielding compound **9** (12.0 mg, *t*_R_ = 38.2 min). Fr14 was divided into Fr14-1 to Fr14-4, where Fr14-4 led to the isolation of compound **10** (13.6 mg, *t*_R_ = 16.2 min). Finally, Fr15 was split into Fr15-1 to Fr15-3, with compound **11** (13.0 mg, *t*_R_ = 17.4 min) being isolated from Fr15-3 via RP-HPLC with a 15–30% acetonitrile in water gradient over 30 min.

#### 4.3.1. Compound **1**

A white amorphous powder; [α${]}_{D}^{25}$ +4.9 (*c* 0.1, MeOH); UV (MeOH) *λ*_max_ (log *ε*): 272 (3.97) nm; ECD (*c* 0.1 mg/mL, MeOH) *λ*_max_ (∆*ε*): 214 (+0.52), 277 (+0.27) nm; HRESIMS *m*/*z* 471.2369 [M + H]^+^ (calcd for C_27_H_35_O_7_, 471.2383); ^1^H and ^13^C NMR data, Table 1.

#### 4.3.2. Compound **2**

A white amorphous powder; [α${]}_{D}^{25}$ +3.4 (*c* 0.1, MeOH); UV (MeOH) *λ*_max_ (log *ε*): 275 (3.59) nm; ECD (*c* 0.1 mg/mL, MeOH) *λ*_max_ (∆*ε*): 217 (+1.80), 283 (+0.52) nm; HRESIMS *m*/*z* 429.2280 [M + H]^+^ (calcd for C_25_H_33_O_6_, 429.2277); ^1^H and ^13^C NMR data, Table 1.

#### 4.3.3. Compound **3**

A white amorphous powder; [α${]}_{D}^{25}$ +46.9 (*c* 0.1, MeOH); UV (MeOH) *λ*_max_ (log *ε*): 274 (3.97) nm; ECD (*c* 0.1 mg/mL, MeOH) *λ*_max_ (∆*ε*): 216 (+5.41), 278 (+1.44) nm; HRESIMS *m*/*z* 509.2156 [M + Na]^+^ (calcd for C_27_H_34_O_8_Na, 509.2151); ^1^H and ^13^C NMR data, Table 1.

### 4.4. Cytotoxic Activity Assay

The cancer cell lines MKN-45, HCT 116, TE-1, and PATU8988T were obtained from Wuhan Pricella Biotechnology Co., Ltd (Wuhan, China). The basis of detection involved the CCK-8 reagent, which includes WST-8. This component is converted into a water-soluble yellow formazan product by mitochondrial dehydrogenases, facilitated by the electron carrier 1-methoxy-5-methylphenazine dimethyl sulfate (1-methoxy PMS). The quantity of formazan generated directly correlates with the number of viable cells.

### 4.5. NMR and ECD Calculations

The conformers were first produced using CREST software (V 2.11) [15,16] and further refined via DFT optimization at the B3LYP/6-31G(d) level with Gaussian 16 (Gaussian, Wallingford, CT, USA) [17]. We focused on conformers that lay within a 10 kcal/mol energy window. Frequency analysis was conducted on each conformer to verify its status as a local minimum. The electronic energies were then recalculated at the higher M062X/6-311+G(2d,p) level. By applying a Boltzmann distribution, we assessed the conformer populations, especially those accounting for more than 2% of the total, for comprehensive examination. Excited state calculations were executed at the Cam-B3LYP/6-311G(d) level using TDDFT, targeting 36 excited states for each conformer [18]. ECD curves were subsequently produced using Multiwfn 3.6 software [19].

## 5. Conclusions

This study deepens our understanding of the chemical diversity and biological activities of secondary metabolites from marine-derived *Talaromyces* strains. The discovery of three novel 8,9-*epi*-chrodrimanin derivatives from *Talaromyces variabilis* M22734 illustrates the vast chemical potential of fungi. The selective cytotoxic effect of compound **3** against MKN-45 cells underscores its potential as a lead compound for new gastrointestinal cancer treatments. This selectivity is particularly crucial compared to the non-selective cytotoxicity of traditional chemotherapeutics like doxorubicin. Furthermore, the minimal activities in compounds **1** and **2** highlight the importance of subtle structural differences in bioactivity, suggesting that minor modifications can lead to significant biological impacts. Future research should focus on optimizing the antitumor properties of these compounds through structural modifications. Additionally, exploring their biosynthetic pathways may provide insights into the genetic and enzymatic mechanisms that microbes use to produce complex molecules, offering potential new targets and strategies for synthetic biology.

## Figures and Tables

**Table 1:** ^1^H (600 MHz) and ^13^C NMR (125 MHz) data of **1**–**3** (chloroform-*d*).

	1		2		3	
Position	*δ*_C_, Type	*δ*_H_, Mult. (*J* in Hz)	*δ*_C_, Type	*δ*_H_, Mult. (*J* in Hz)	*δ*_C_, Type	*δ*_H_, Mult. (*J* in Hz)
1a-*β*	44.2, CH_2_	2.38 (m)	40.4, CH_2_	2.06 (ddd, 12.9, 6.4, 2.7 Hz)	46.3, CH_2_	2.34 (dd, 12.1, 6.2 Hz)
1b-*α*	44.2, CH_2_	1.57 (d, 13.2 Hz)	40.4, CH_2_	1.47 (m)	46.3, CH_2_	1.51 (d, 4.8 Hz)
2a-*β*	71.1, CH	5.68 (dd, 13.2, 6.1 Hz)	34.0, CH_2_	2.85 (ddd, 15.2, 13.7, 6.4 Hz)	71.2, CH	5.79 (dd, 13.3, 6.1 Hz)
2b-*α*			34.0, CH_2_	2.36 (ddd, 15.2, 5.0, 2.7 Hz)		
3	208.0, C	-	215.1, C	-	207.7, C	-
4	48.5, C	-	48.8, C	-	49.2, C	-
5	56.6, CH	1.47 (dd, 11.9, 2.2 Hz)	56.5, CH	1.44 (m)	56.9, CH	1.39 (m)
6a-*α*	19.9, CH_2_	1.78 (dq, 13.2, 3.3 Hz)	68.5, CH	4.61 (brs)	68.4, CH	4.62 (m)
6b-*β*	19.9, CH_2_	1.63 (m, 1H)				
7a-*β*	40.1, CH_2_	2.16 (m)	48.3, CH_2_	2.28 (dd, 14.1, 3.1 Hz)	48.3, CH_2_	2.28 (dd, 14.3, 3.0 Hz)
7b-*α*	40.1, CH_2_	1.72 (m)	48.3, CH_2_	1.93 (dd, 14.1, 3.5 Hz)	48.3, CH_2_	1.93 (dd, 14.2, 3.7 Hz)
8	77.2, C	-	77.0, C	-	76.9, C	-
9	51.2, CH	1.70 (m)	51.7, CH	1.70 (dd, 13.1, 5.0 Hz)	51.7, CH	1.73 (dd, 12.9, 5.1 Hz)
10	37.5, C	-	36.8, C	-	37.7, C	-
11a-*α*	19.6, CH_2_	2.53 (dd, 15.8, 5.1 Hz)	19.4, CH_2_	2.58 (dd, 15.8, 5.0 Hz)	19.5, CH_2_	2.57 (dd, 15.8, 5.1 Hz)
11b-*β*	19.6, CH_2_	2.35 (m)	19.4, CH_2_	2.44 (dd, 15.8, 13.1 Hz)	19.5, CH_2_	2.48 (dd, 15.8, 13.0 Hz)
12	21.0, CH_3_	1.25 (s)	22.2, CH_3_	1.48 (s, 3H)	22.3, CH_3_	1.48 (s, 3H)
13-*α*	25.0, CH_3_	1.18 (s)	25.5, CH_3_	1.22 (s, 3H)	24.9, CH_3_	1.25 (s, 3H)
14-*β*	21.2, CH_3_	1.19 (s)	23.9, CH_3_	1.45 (s, 3H)	23.2, CH_3_	1.55 (s, 3H)
15	15.3, CH_3_	1.30 (s)	15.7, CH_3_	1.50 (s, 3H)	16.9, CH_3_	1.67 (s, 3H)
16	170.2, C	-			170.1, C	-
17	20.8, CH_3_	2.17 (s)			20.8, CH_3_	2.18 (s, 3H)
1′	101.9, C	-	101.8, C	-	101.9, C	-
2′	139.1, C	-	138.9, C	-	139.0, C	-
3′	110.1, C	-	110.8, C	-	110.3, C	-
4′	162.4, C	-	162.4, C	-	162.4, C	-
5′	103.5, CH	6.29 (s)	103.5, CH	6.29 (s, 1H)	103.5, CH	6.29 (s, 1H)
6′	160.1, C	-	159.9, C	-	159.7, C	-
7′a	31.4, CH_2_	2.93 (dd, 16.6, 3.4 Hz)	31.4, CH_2_	2.94 (dd, 16.7, 3.4 Hz)	31.4, CH_2_	2.95 (dd, 16.7, 3.4 Hz)
7′b	31.4, CH_2_	2.63 (dd, 16.7, 11.5 Hz)	31.4, CH_2_	2.64 (dd, 16.7, 11.5 Hz)	31.4, CH_2_	2.64 (dd, 16.7, 11.4 Hz)
8′	74.6, CH	4.64 (dqd, 12.6, 6.3, 3.4 Hz)	74.6, CH	4.65 (ddp, 12.6, 6.3, 3.2 Hz)	74.6, CH	4.64 (m)
9′	20.9, CH_3_	1.55 (d, 6.3 Hz)	20.9, CH_3_	1.55 (d, 6.3 Hz)	20.9, CH_3_	1.56 (d, 6.3 Hz)
10′	170.0, C	-	170.1, C	-	170.0, C	-
31	OH	11.11 (s)	OH	11.11 (s)	OH	11.12 (s)

**Table 2:** The inhibition rates of compounds **1**–**3** against four different gastrointestinal cancer cell lines at a concentration of 20 μM.

Compounds	Cell Inhibition ± SD (%)			
	MKN-45	HCT 116	TE-1	PATU8988T
**1**	16.2 ± 1.6%	1.2 ± 2.2%	4.1 ± 2.2%	2.6 ± 1.8%
**2**	14.7 ± 2.1%	−0.1 ± 2.1%	2.2 ± 4.8%	3.4 ± 0.4%
**3**	55.3 ± 2.1%	4.6 ± 4.1%	5.1 ± 3.3%	0.8 ± 1.3%
Dox	79.4 ± 0.7%	76.9 ± 0.5%	88.7 ± 1.9%	94.9 ± 0.7%

## Data Availability

The original contributions presented in the study are included in the article/Supplementary Material, further inquiries can be directed to the corresponding author/s.

## Supplementary Materials

The following supporting information can be downloaded via this link: https://www.mdpi.com/article/10.3390/md22060274/s1, Figures S1–S24; Tables S1–S13.
