# Supplementary material for: Cytotoxic Pentaketide-Sesquiterpenes from the Marine-Derived Fungus Talaromyces variabilis M22734
Source: Mar Drugs. 2024 Jun 13;22(6):274. doi: 10.3390/md22060274 (PMC11204578; doi:10.3390/md22060274)
Supplement: Supplementary file 1 [file marinedrugs-22-00274-s001.zip › marinedrugs-3059767-supplementary.pdf]

Cytotoxic Pentaketide-Sesquiterpenes from the Marine Derived Fungus *Talaromyces variabilis* M22734

Lingzhi Tang [1,†], Jinmei Xia [2,†], Zhongwei Chen [1,†], Xiaohui Wu [1,3], Guangyu Li [2], Qiliang Lai [2], Zongze Shao [2], Weiyi Wang [2,*], Xuan Hong [1,3*]

[1] Xiamen Key Laboratory of Marine Medicinal Natural Products Resources, Xiamen Medical College, Xiamen 361023, China

[2] Key Laboratory of Marine Biogenetic Resources, Third Institute of Oceanography, Ministry of Natural Resources, Xiamen 361005, China

[3] School of Pharmacy, Fujian Medical University, Fuzhou 350122, China

Table of Contents

Figure S1. MS spectrum of 1 3

Figure S2. ^{1}H NMR spectrum of 1 3

Figure S3. ^{13}C NMR spectrum of 1 4

Figure S4. DEPT spectrum of 1 5

Figure S5. HSQC spectrum of 1 6

Figure S6. HMBC spectrum of 1 7

Figure S7. COSY spectrum of 1 8

Figure S8. NOESY spectrum of 1 9

Figure S9. MS spectrum of 2 9

Figure S10. ^{1}H NMR spectrum of 2 10

Figure S11. ^{13}C NMR spectrum of 2 11

Figure S12. DEPT spectrum of 2 12

Figure S13. HSQC spectrum of 2 13

Figure S14. HMBC spectrum of 2 14

Figure S15. COSY spectrum of 2 15

Figure S16. NOESY spectrum of 2 16

Figure S17. MS spectrum of 3 16

Figure S18. ^{1}H NMR spectrum of 3 17

Figure S19. ^{13}C NMR spectrum of 3 18

Figure S20. DEPT spectrum of 3 19

Figure S21. HSQC spectrum of 3 20

Figure S22. HMBC spectrum of 3 21

Figure S23. COSY spectrum of 3 22

Figure S24. NOESY spectrum of 3 23
Table S1. Conformational analysis of the B3LYP/6-31G(d) optimized conformers of (5R,6R,8R,9R,10S,8'R)-2 in the gas phase (T=298.15 K) 23
Table S2. Atomic coordinates (Å) of (5R,6R,8R,9R,10S,8'R)-2-1 obtained at the B3LYP/6-31G(d) level of theory in the gas phase. 23
Table S3. Atomic coordinates (Å) of (5R,6R,8R,9R,10S,8'R)-2-2 obtained at the B3LYP/6-31G(d) level of theory in the gas phase. 24
Table S4. Atomic coordinates (Å) of (5R,6R,8R,9R,10S,8'R)-2-3 obtained at the B3LYP/6-31G(d) level of theory in the gas phase. 25
Table S5. Atomic coordinates (Å) of (5R,6R,8R,9R,10S,8'R)-2-4 obtained at the B3LYP/6-31G(d) level of theory in the gas phase. 25
Table S6. Atomic coordinates (Å) of (5R,6R,8R,9R,10S,8'R)-2-5 obtained at the B3LYP/6-31G(d) level of theory in the gas phase. 26
Table S7. Atomic coordinates (Å) of (5R,6R,8R,9R,10S,8'R)-2-6 obtained at the B3LYP/6-31G(d) level of theory in the gas phase. 27
Table S8. Key transitions, oscillator strengths, and rotatory strengths in the ECD spectrum of conformer (5R,6R,8R,9R,10S,8'R)-2-1 at the CAM-B3LYP/6-311G(d) level of theory in MeOH with IEFPCM solvent model 28
Table S9. Key transitions, oscillator strengths, and rotatory strengths in the ECD spectrum of conformer (5R,6R,8R,9R,10S,8'R)-2-2 at the CAM-B3LYP/6-311G(d) level of theory in MeOH with IEFPCM solvent model 29
Table S10. Key transitions, oscillator strengths, and rotatory strengths in the ECD spectrum of conformer (5R,6R,8R,9R,10S,8'R)-2-3 at the CAM-B3LYP/6-311G(d) level of theory in MeOH with IEFPCM solvent model 31
Table S11. Key transitions, oscillator strengths, and rotatory strengths in the ECD spectrum of conformer (5R,6R,8R,9R,10S,8'R)-2-4 at the CAM-B3LYP/6-311G(d) level of theory in MeOH with IEFPCM solvent model 32
Table S12. Key transitions, oscillator strengths, and rotatory strengths in the ECD spectrum of conformer (5R,6R,8R,9R,10S,8'R)-2-5 at the CAM-B3LYP/6-311G(d) level of theory in MeOH with IEFPCM solvent model 34
Table S13. Key transitions, oscillator strengths, and rotatory strengths in the ECD spectrum of conformer (5R,6R,8R,9R,10S,8'R)-2-6 at the CAM-B3LYP/6-311G(d) level of theory in MeOH with IEFPCM solvent model 36

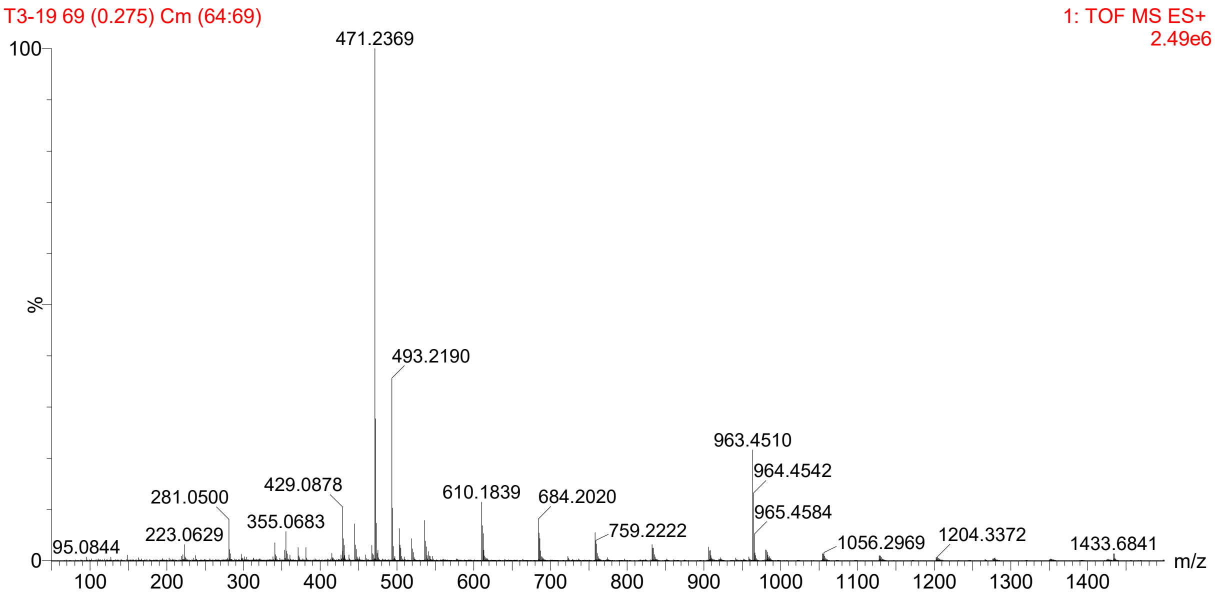

Figure S1. MS spectrum of **1**

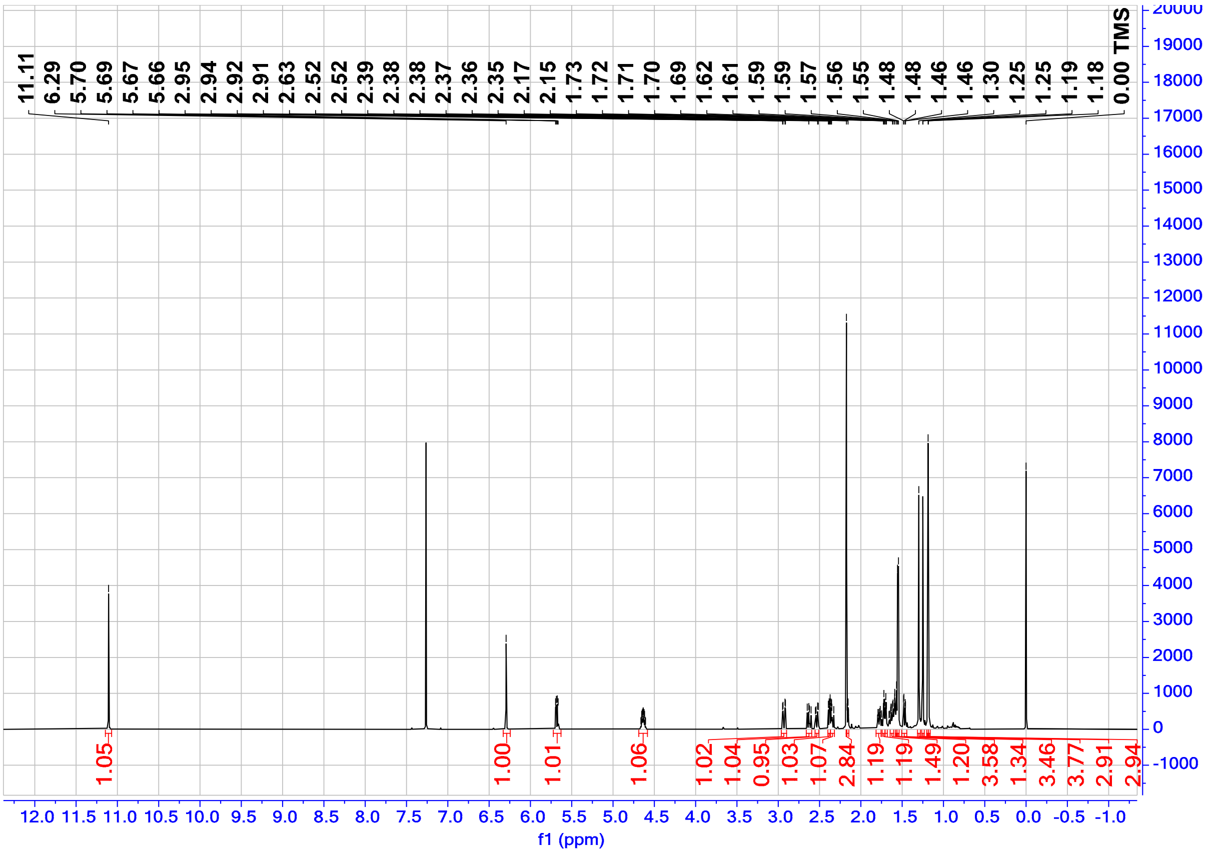

Figure S2. ^{1}H NMR spectrum of **1**

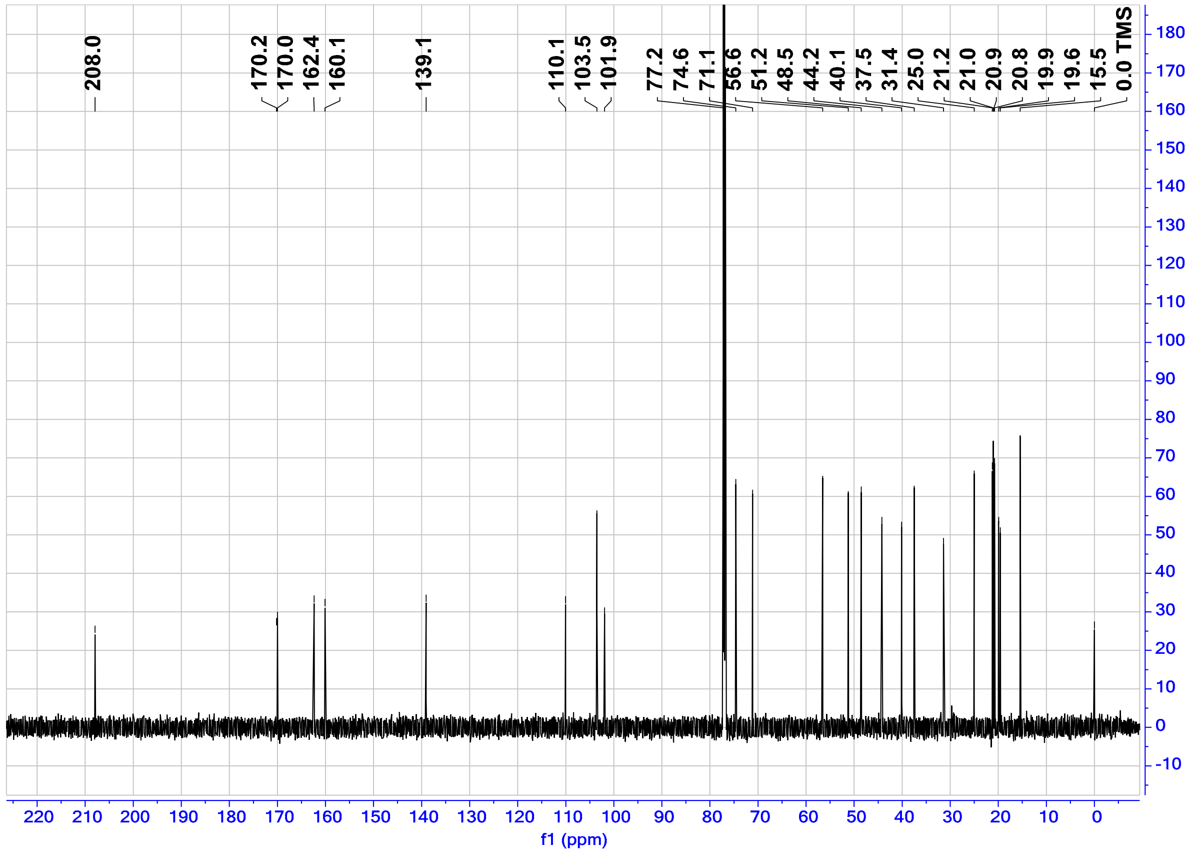

Figure S3. ^{13}C NMR spectrum of **1**

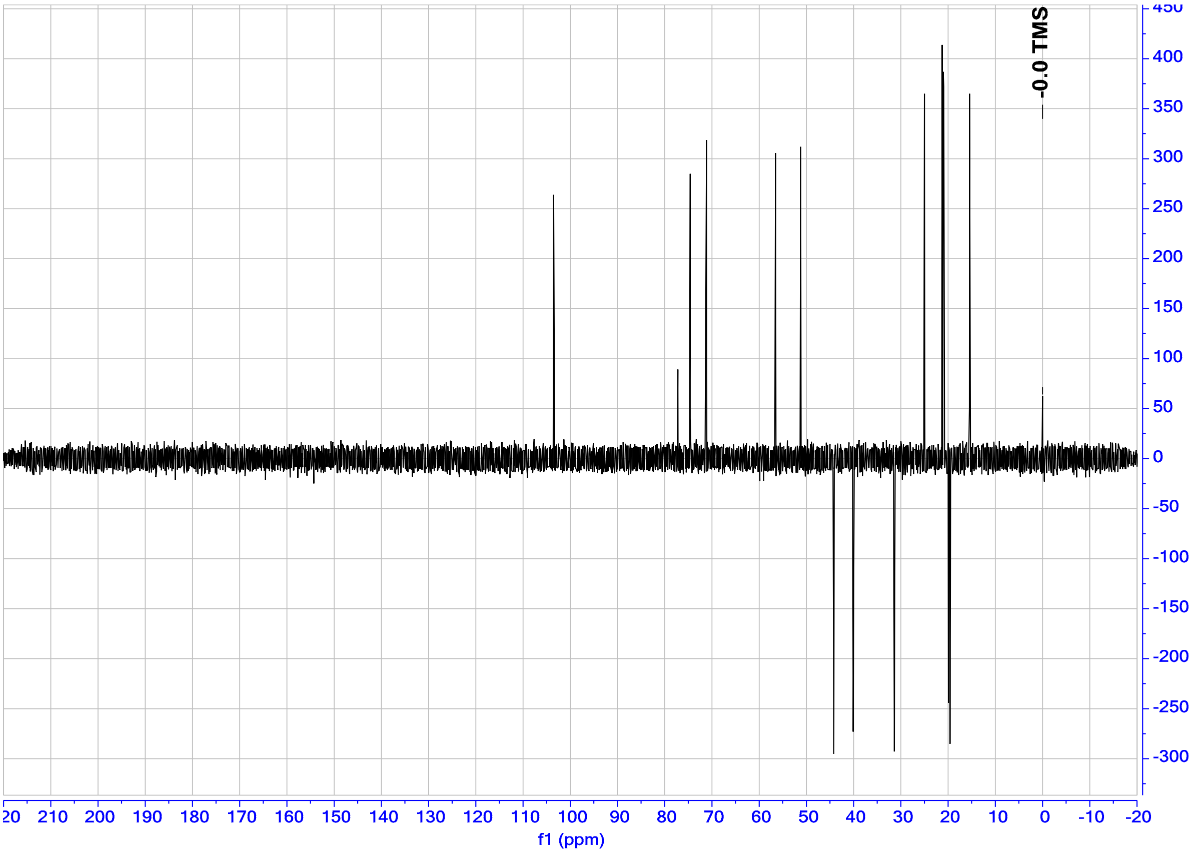

Figure S4. DEPT spectrum of **1**

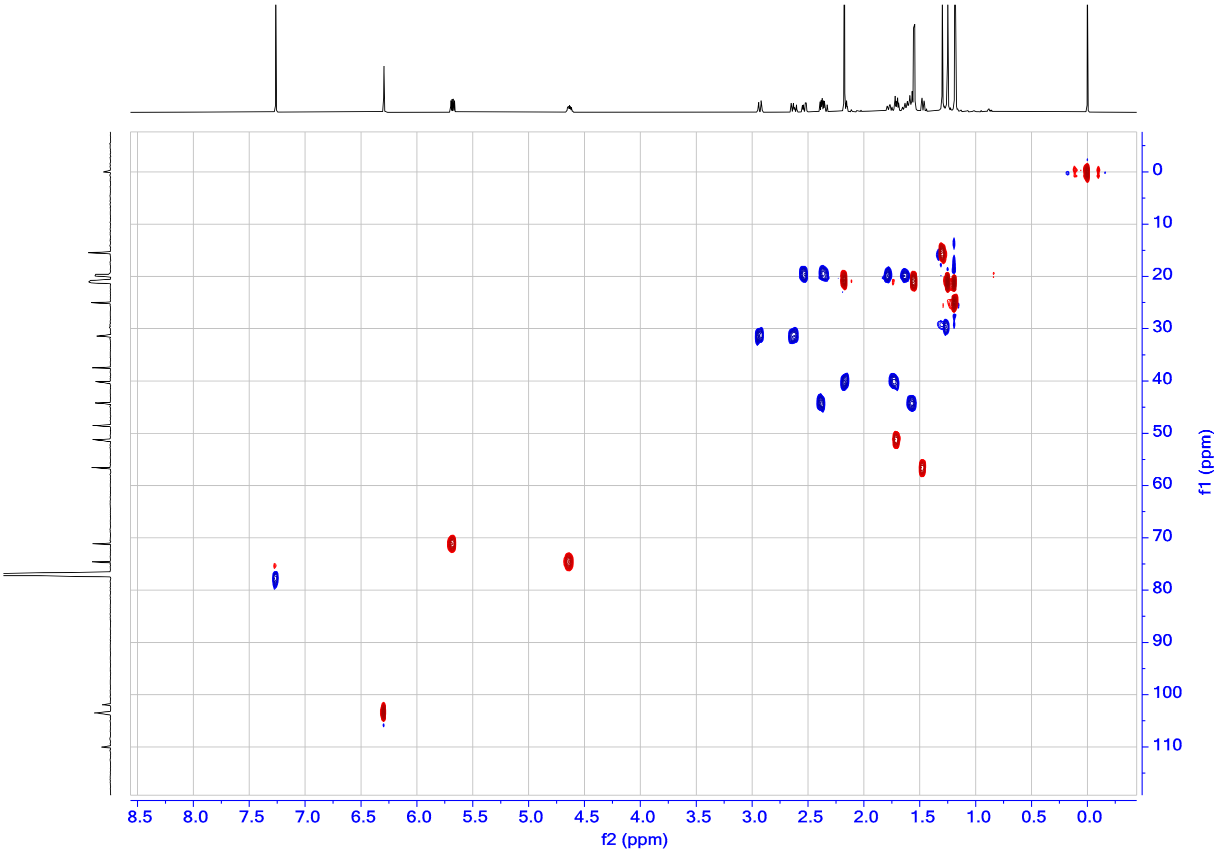

Figure S5. HSQC spectrum of **1**

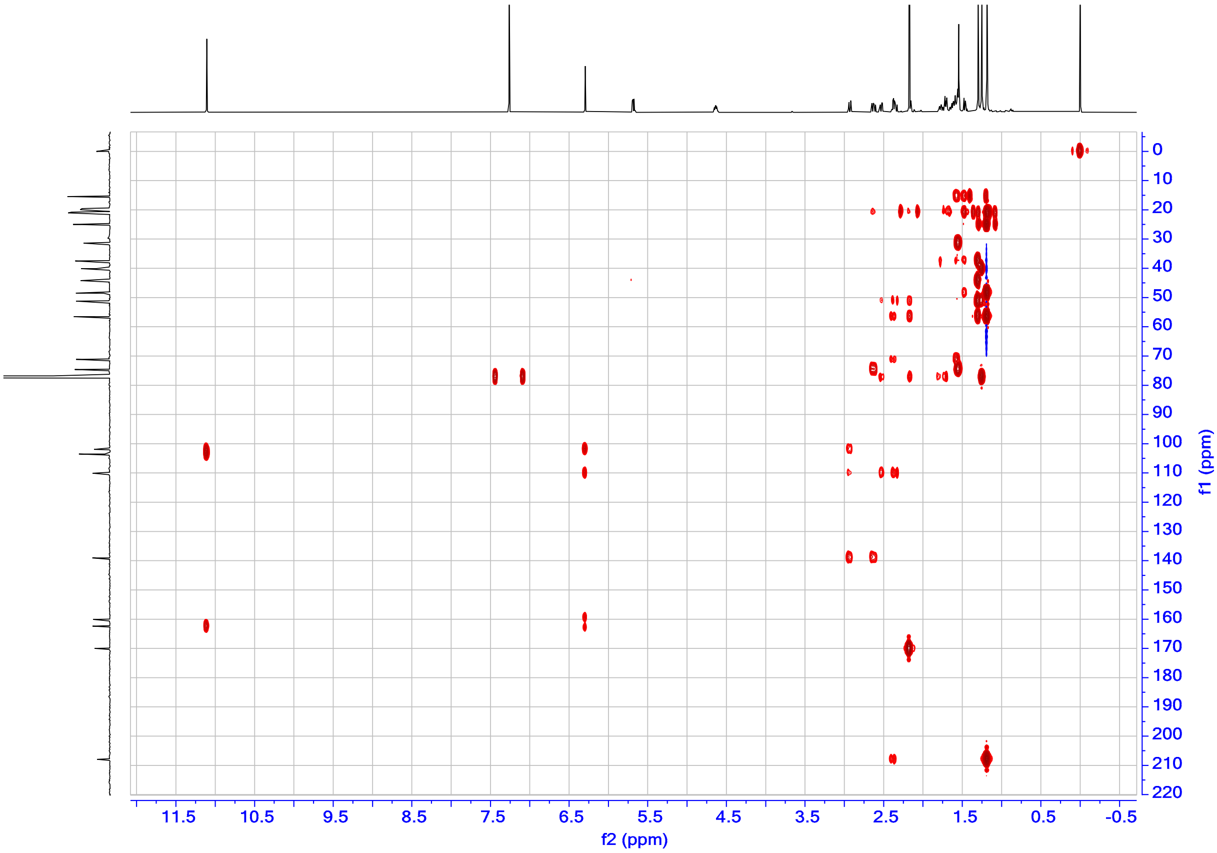

Figure S6. HMBC spectrum of **1**

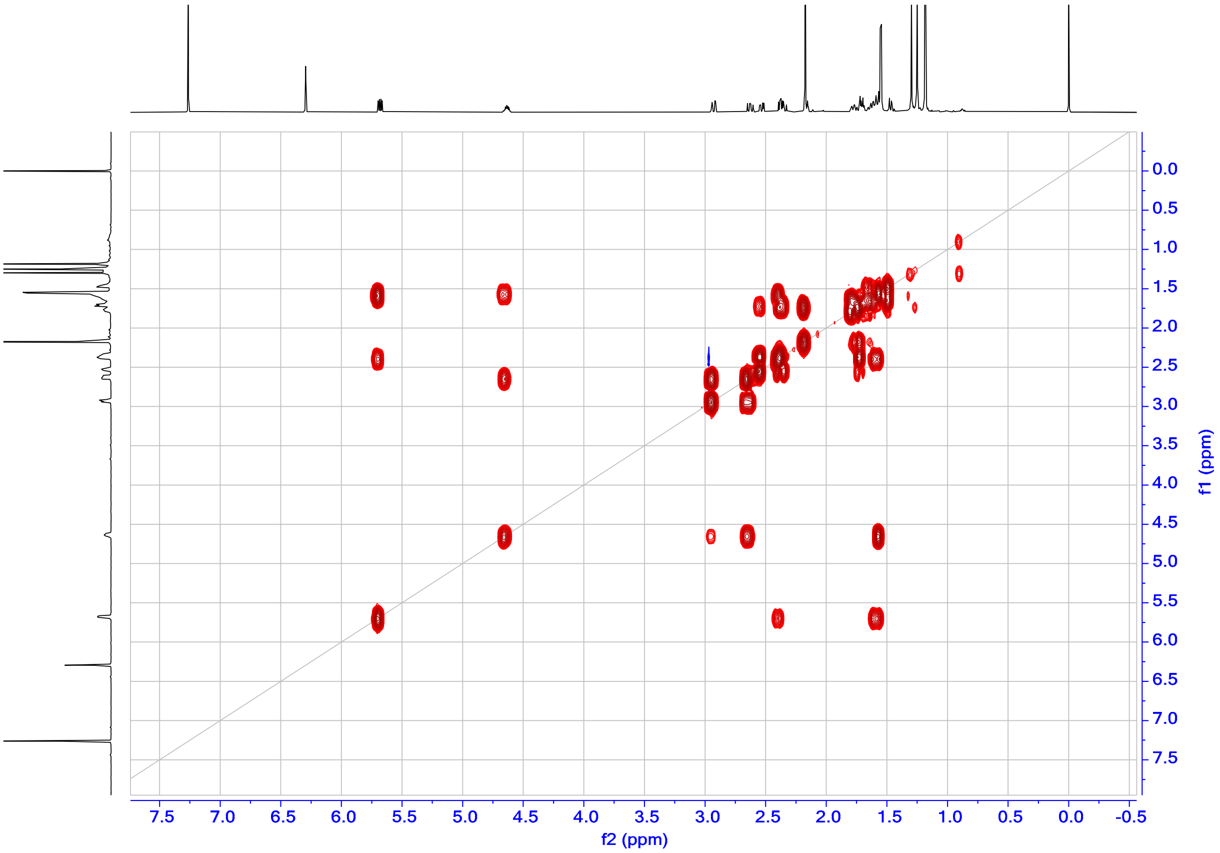

Figure S7. COSY spectrum of **1**

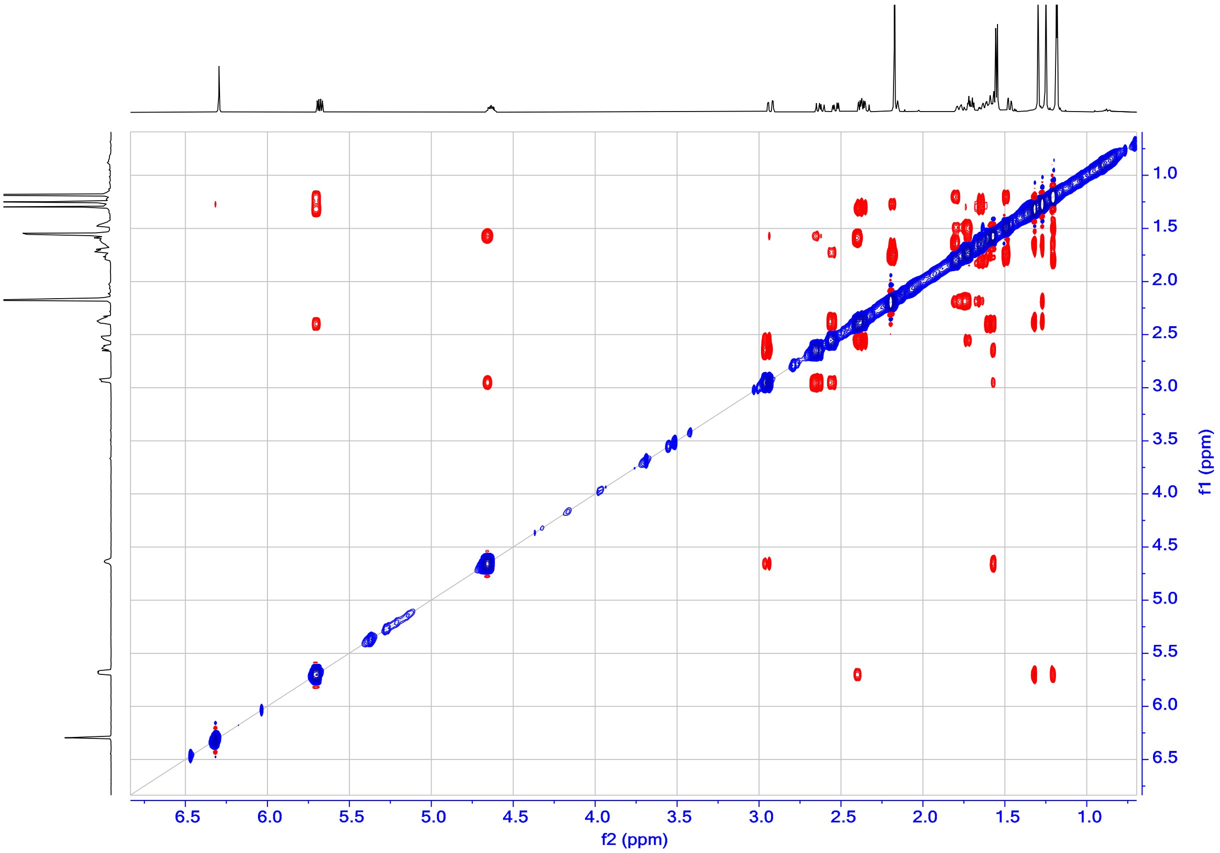

Figure S8. NOESY spectrum of **1**

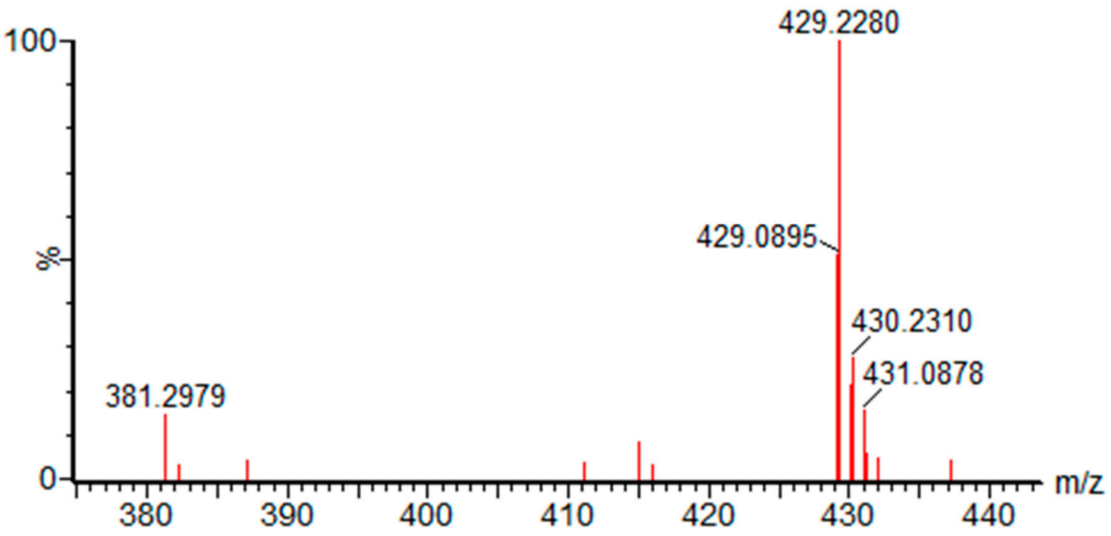

Figure S9. MS spectrum of **2**

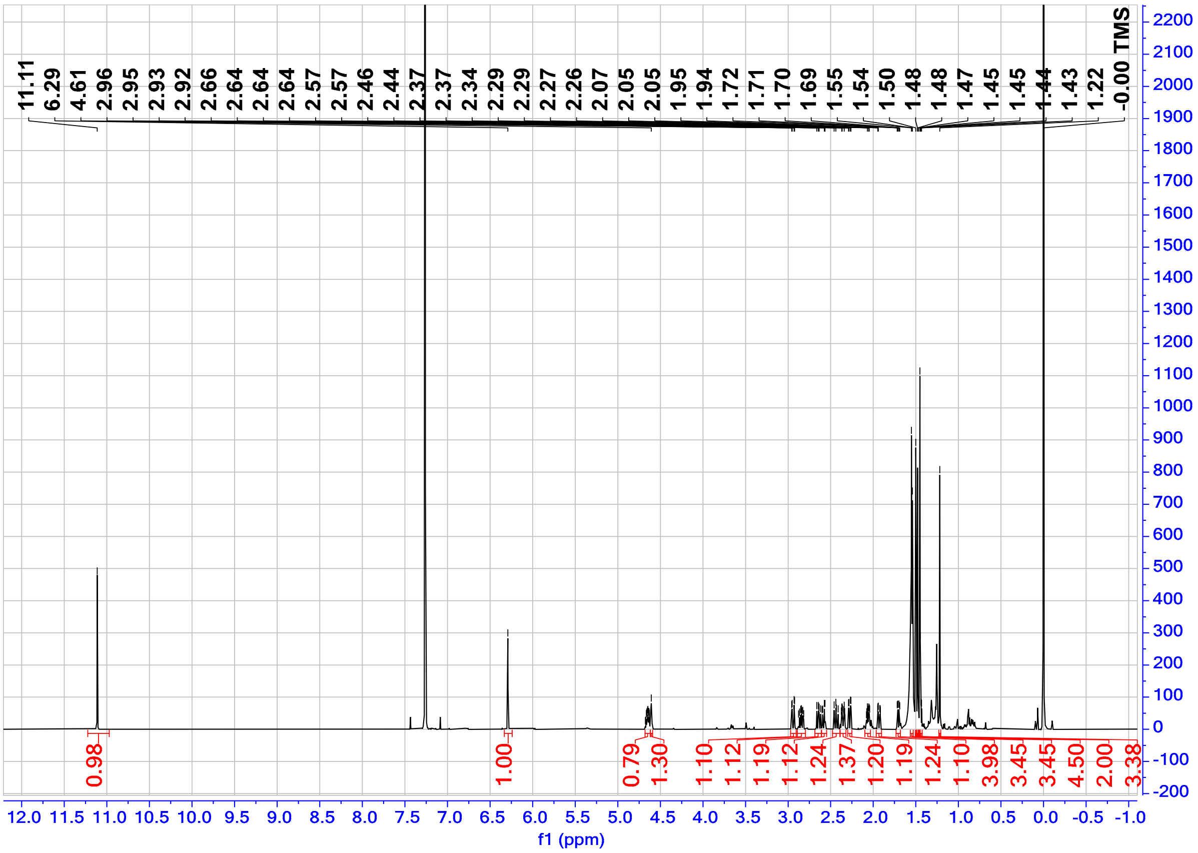

Figure S10. ^{1}H NMR spectrum of **2**

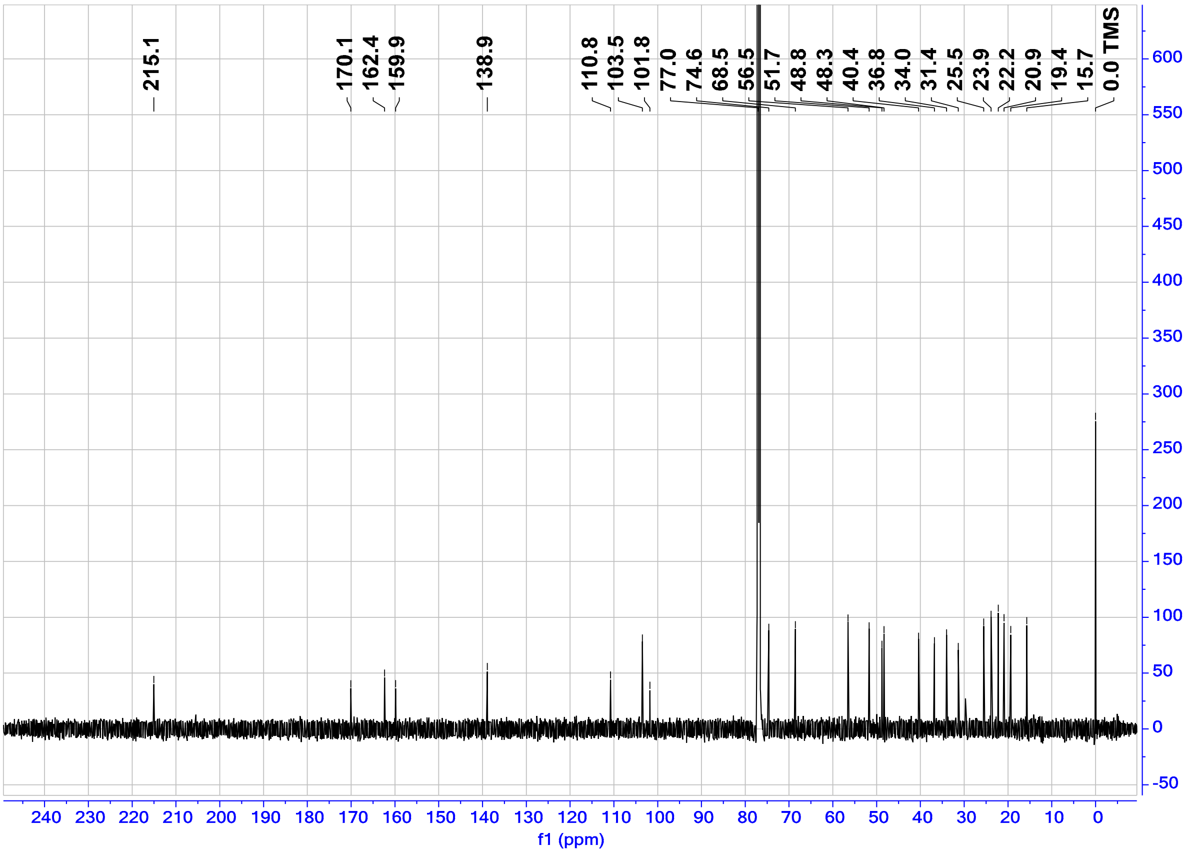

Figure S11. ^{13}C NMR spectrum of **2**

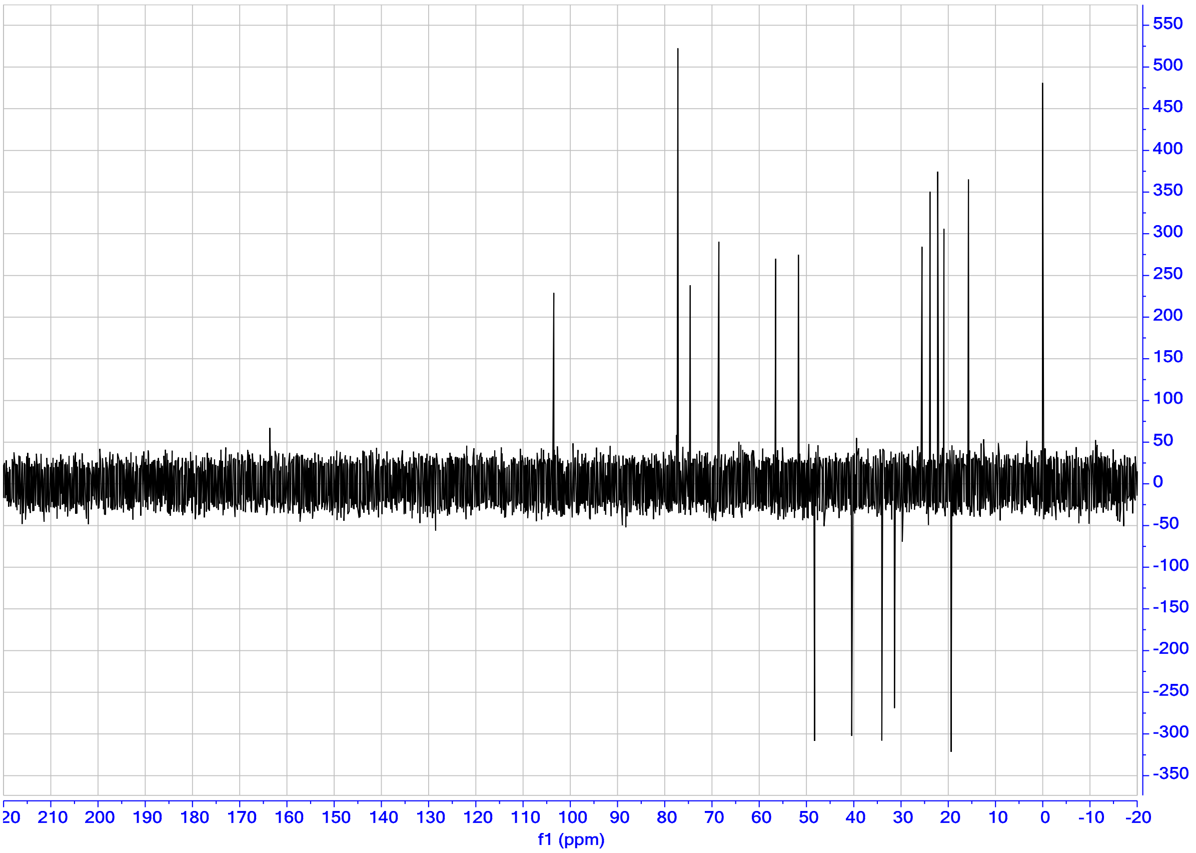

Figure S12. DEPT spectrum of **2**

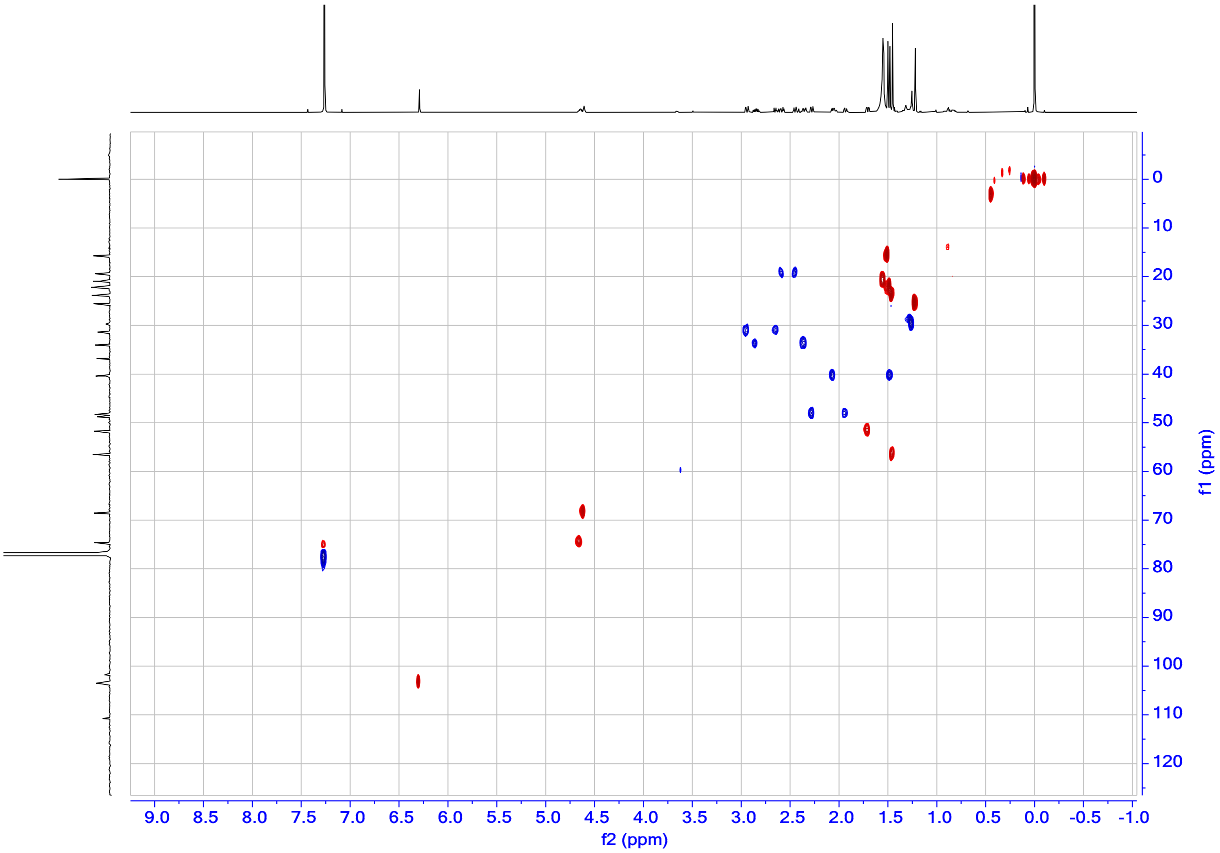

Figure S13. HSQC spectrum of **2**

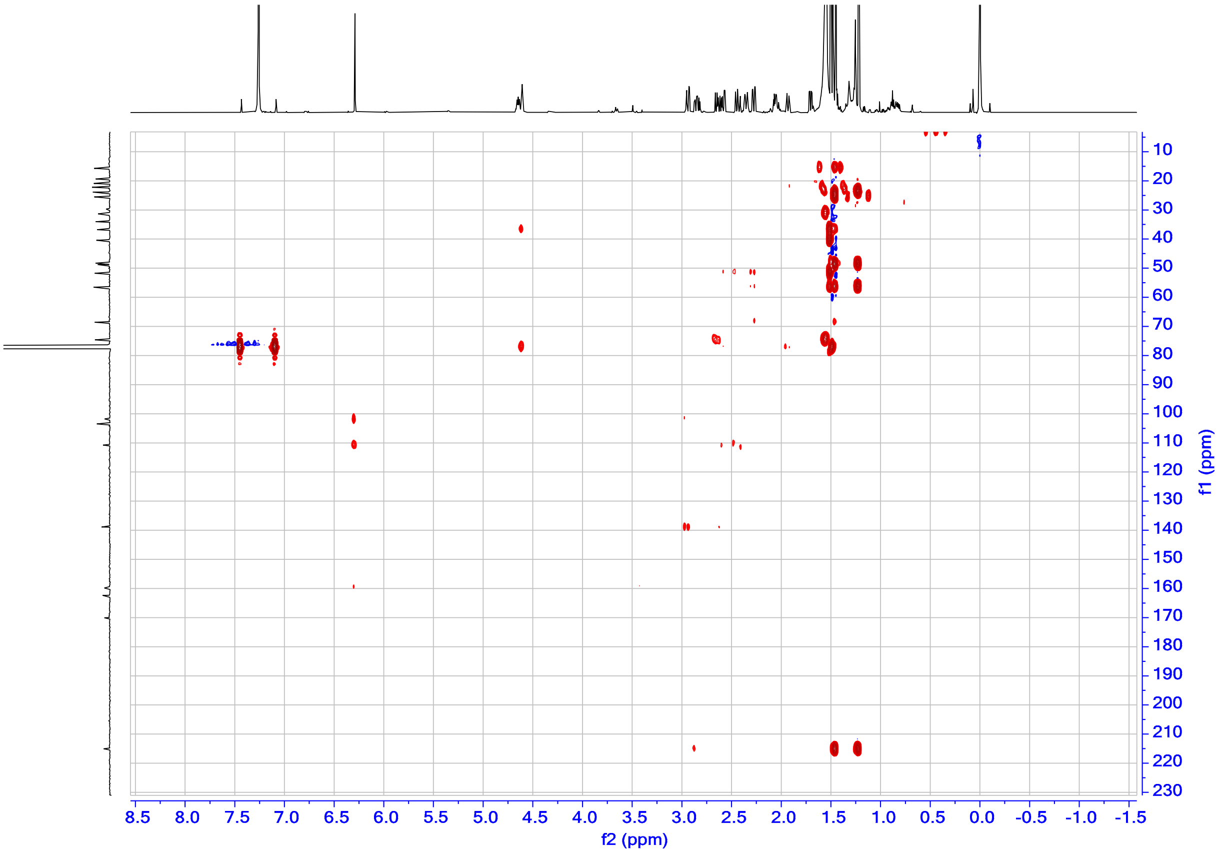

Figure S14. HMBC spectrum of **2**

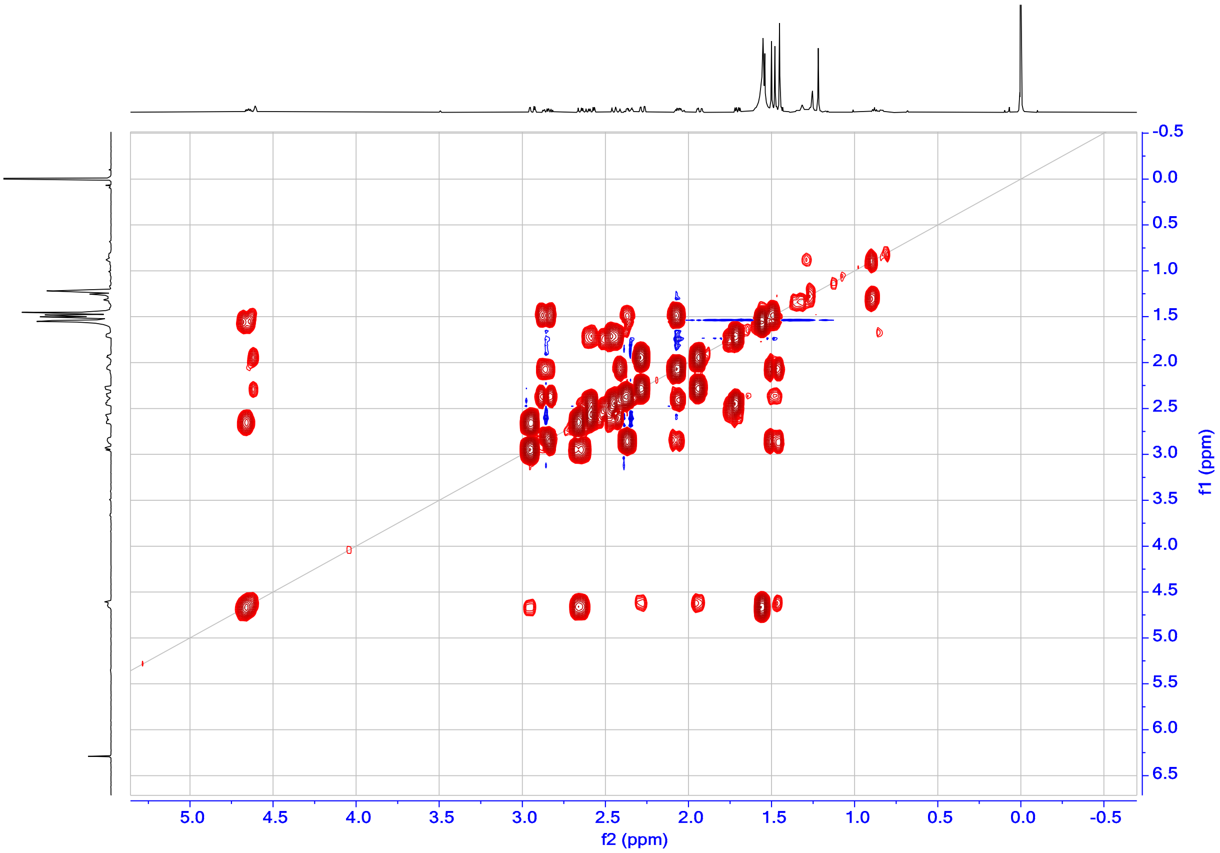

Figure S15. COSY spectrum of **2**

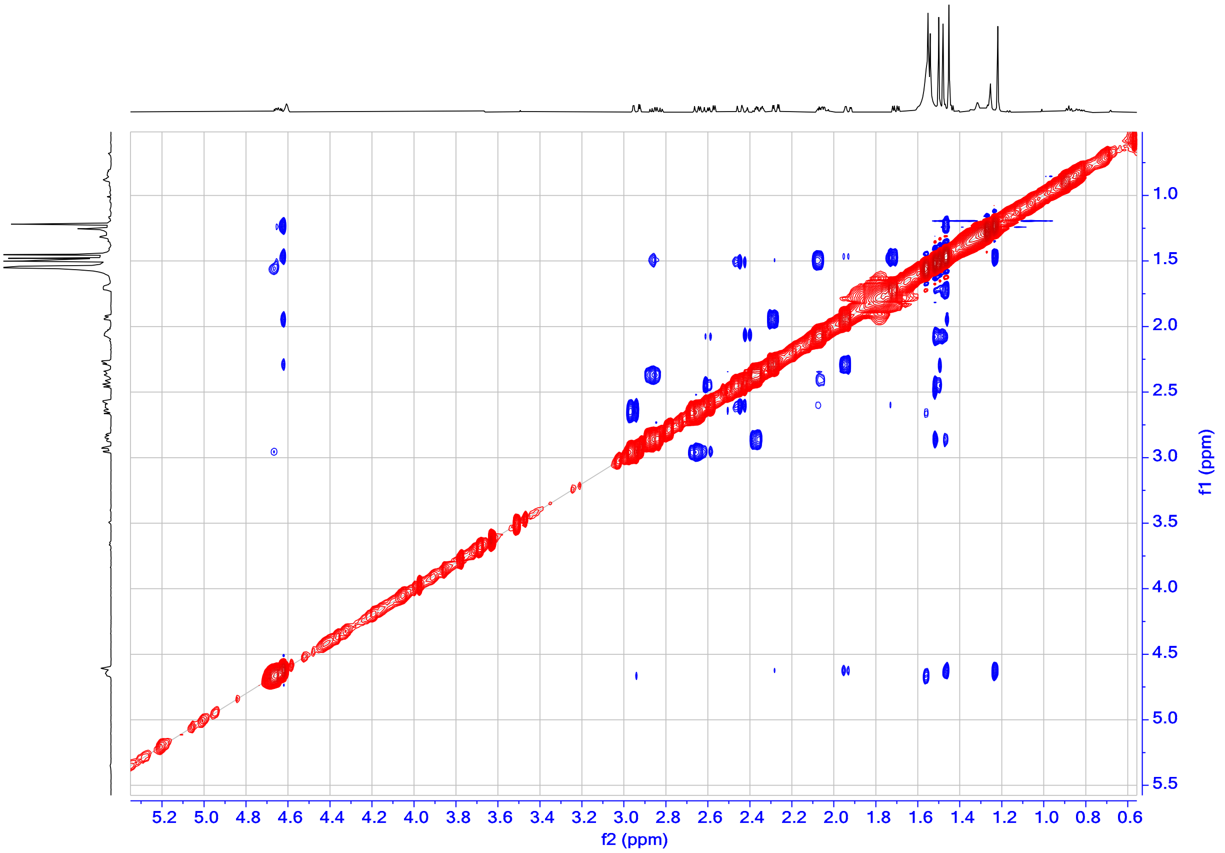

Figure S16. NOESY spectrum of **2**

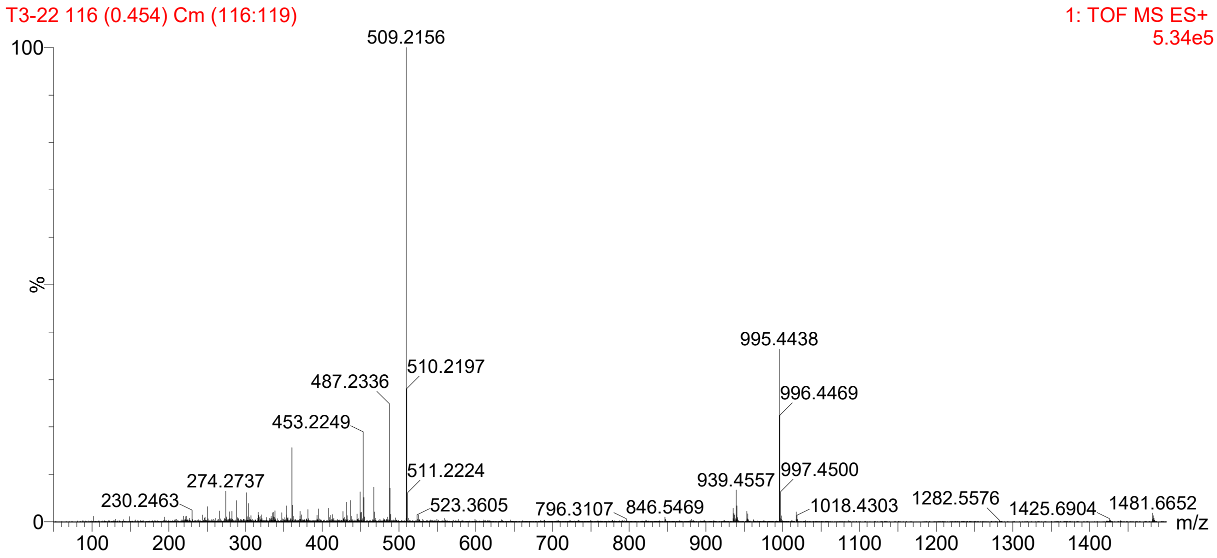

Figure S17. MS spectrum of **3**

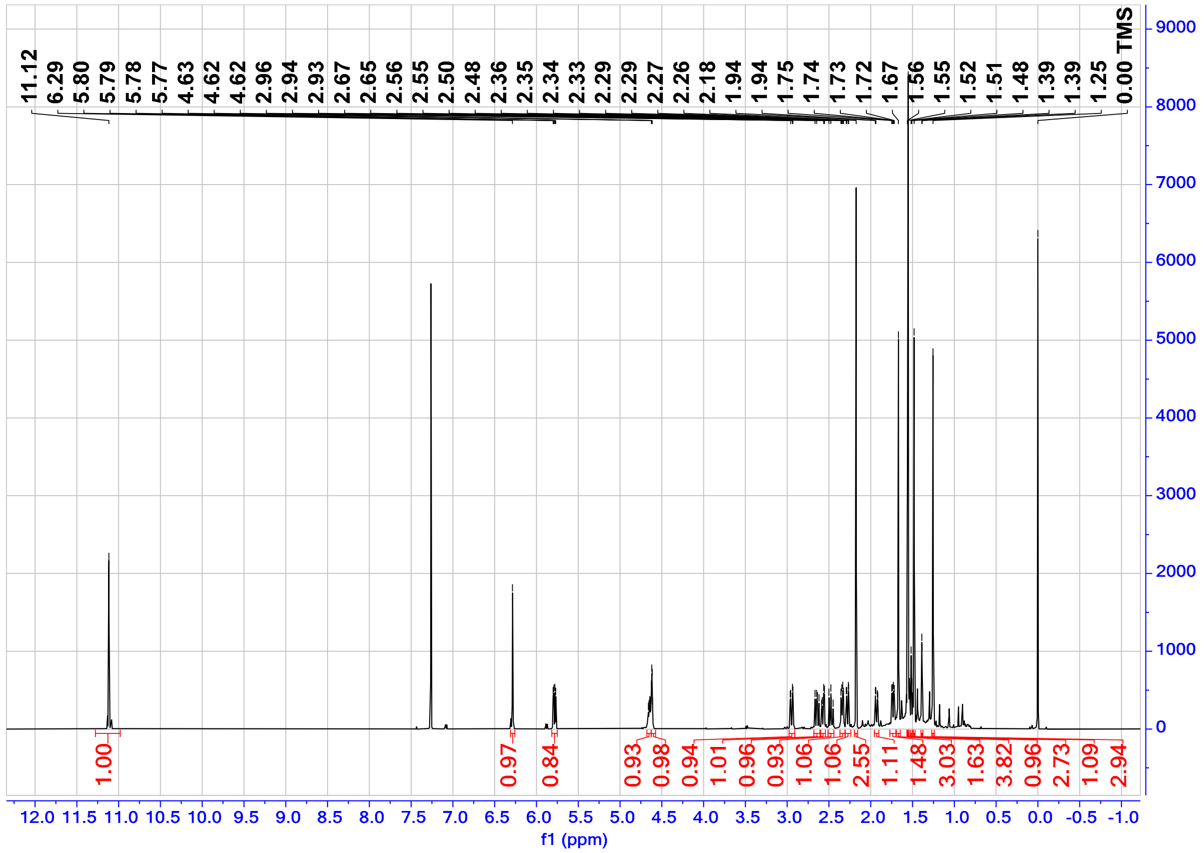

Figure S18. ^{1}H NMR spectrum of **3**

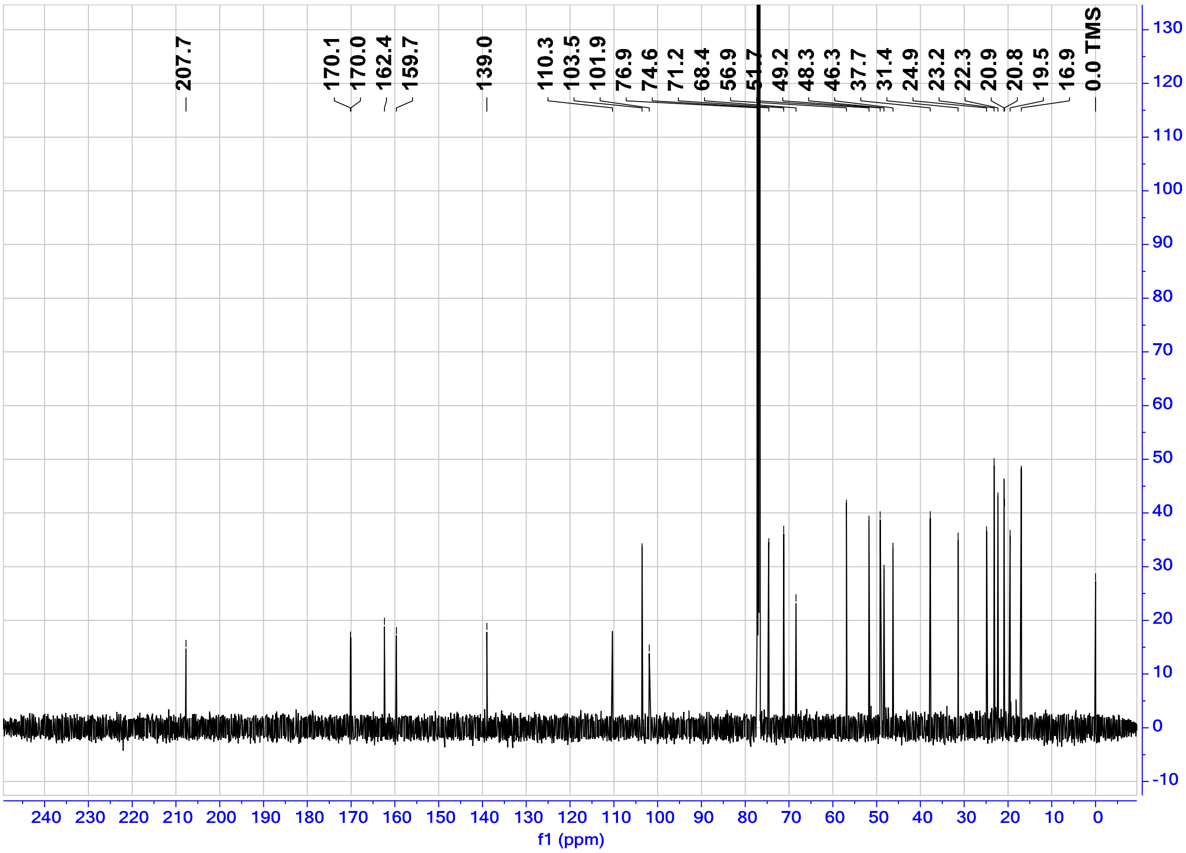

Figure S19. ^{13}C NMR spectrum of **3**

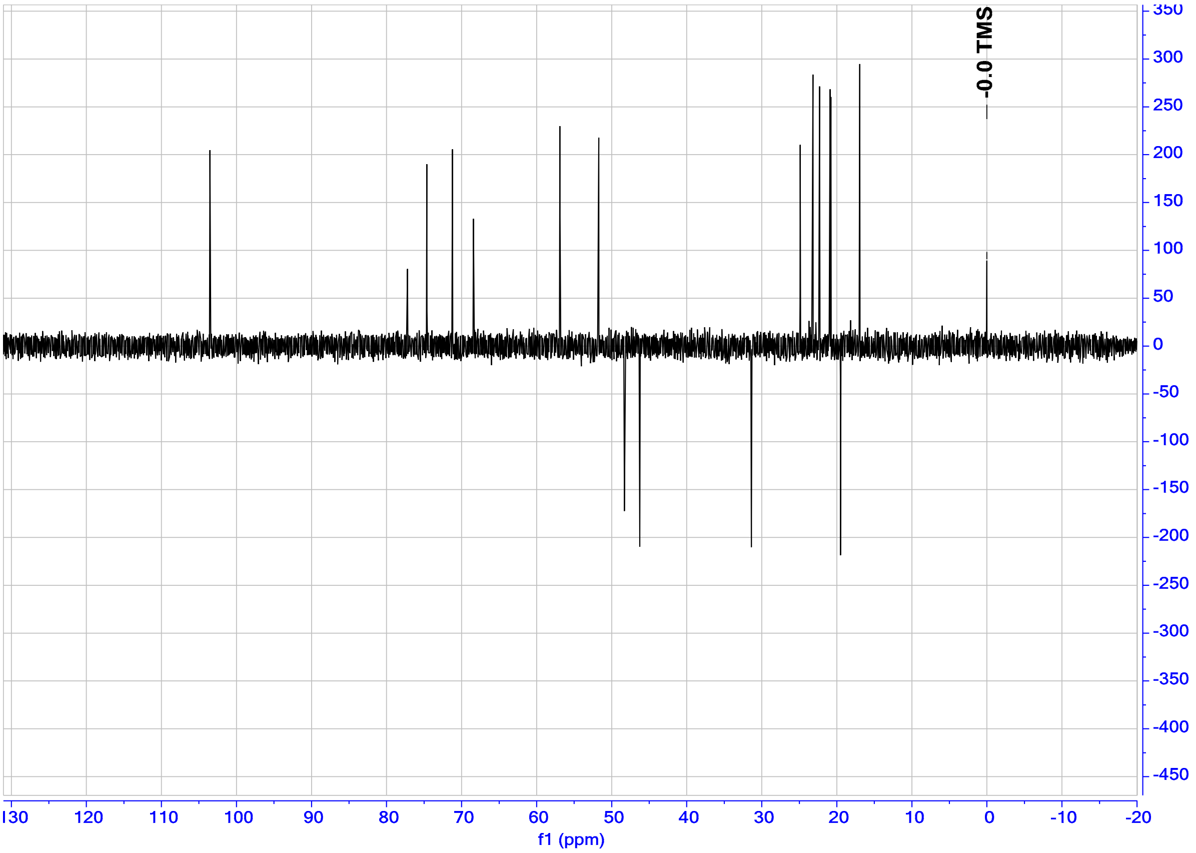

Figure S20. DEPT spectrum of **3**

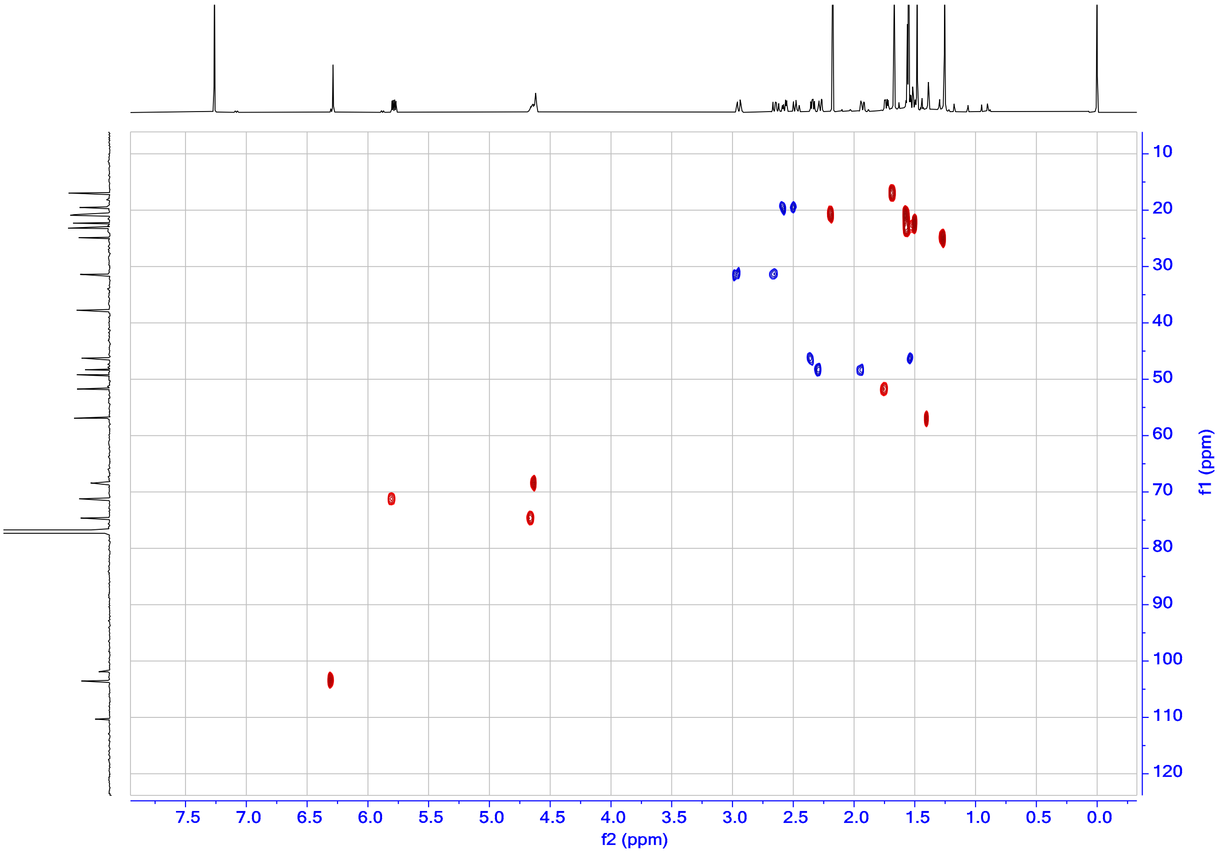

Figure S21. HSQC spectrum of **3**

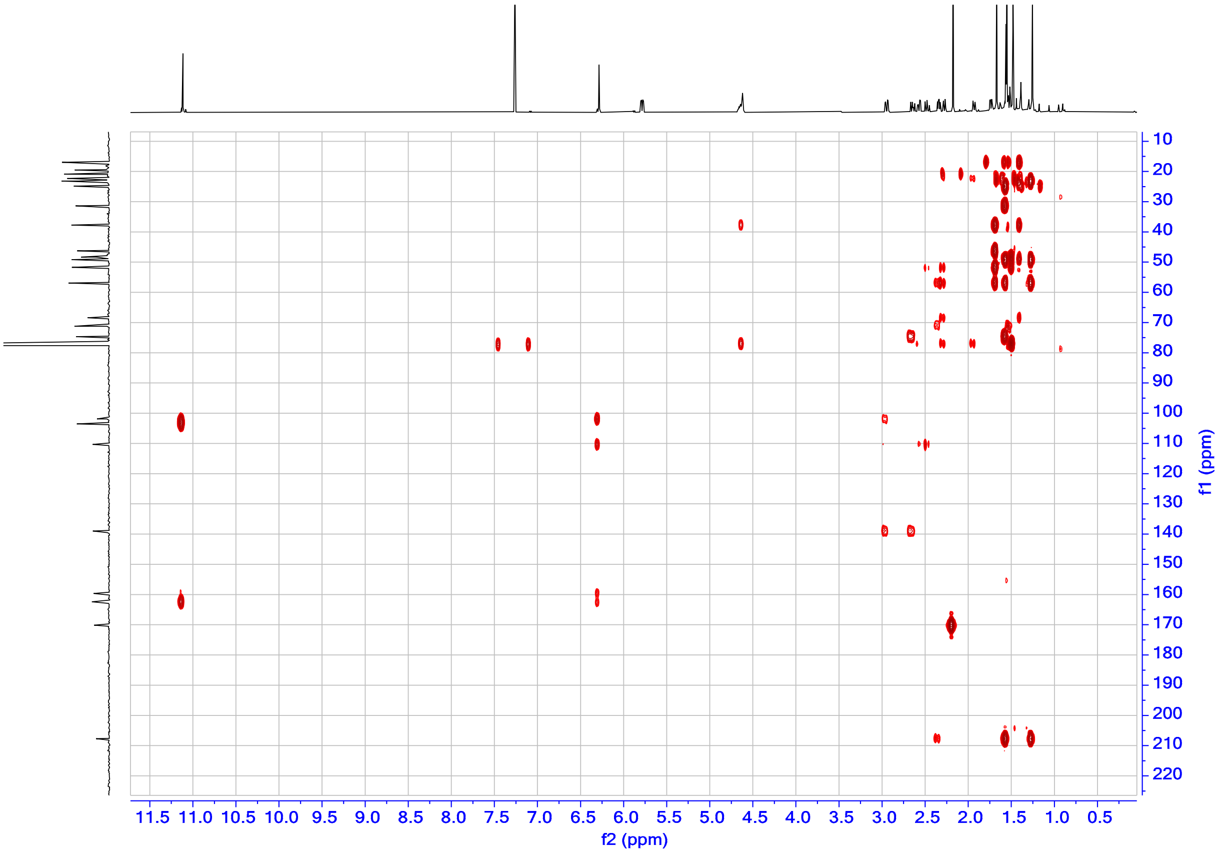

Figure S22. HMBC spectrum of **3**

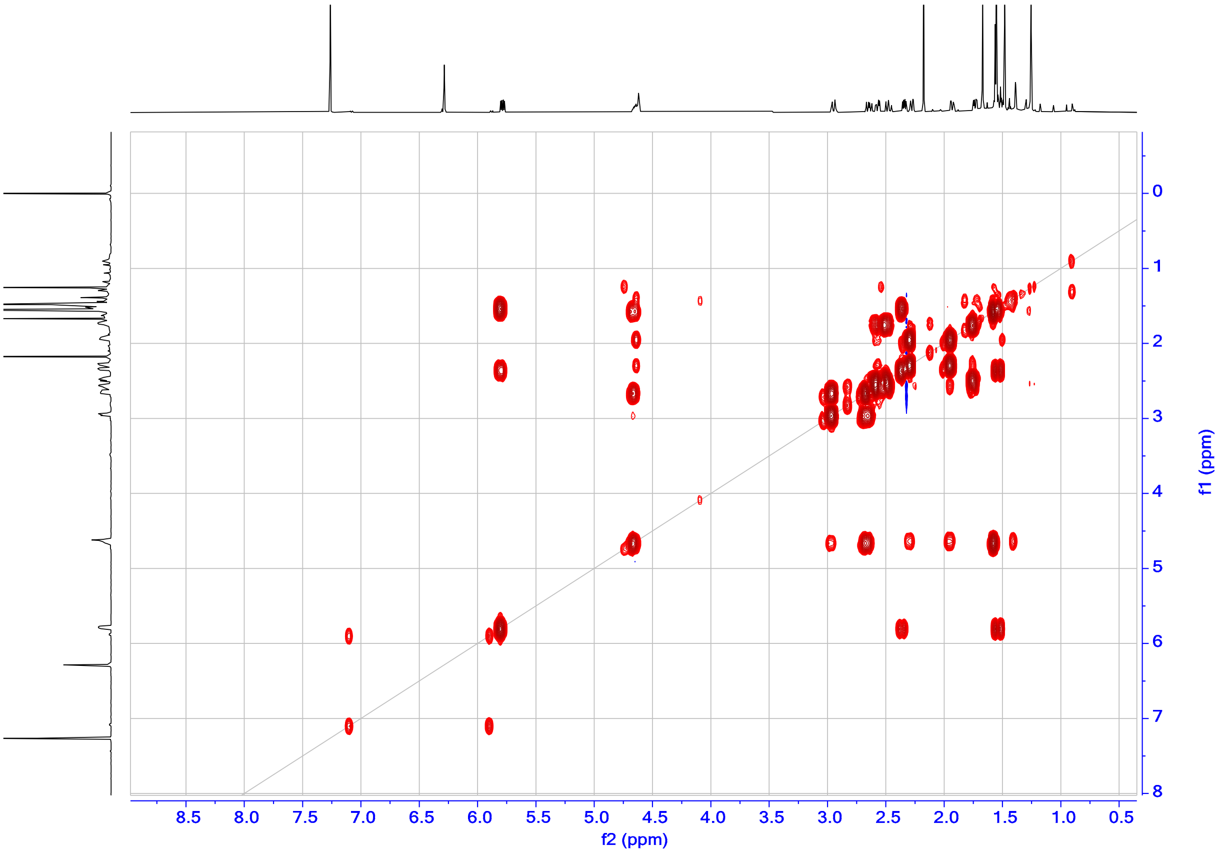

Figure S23. COSY spectrum of **3**

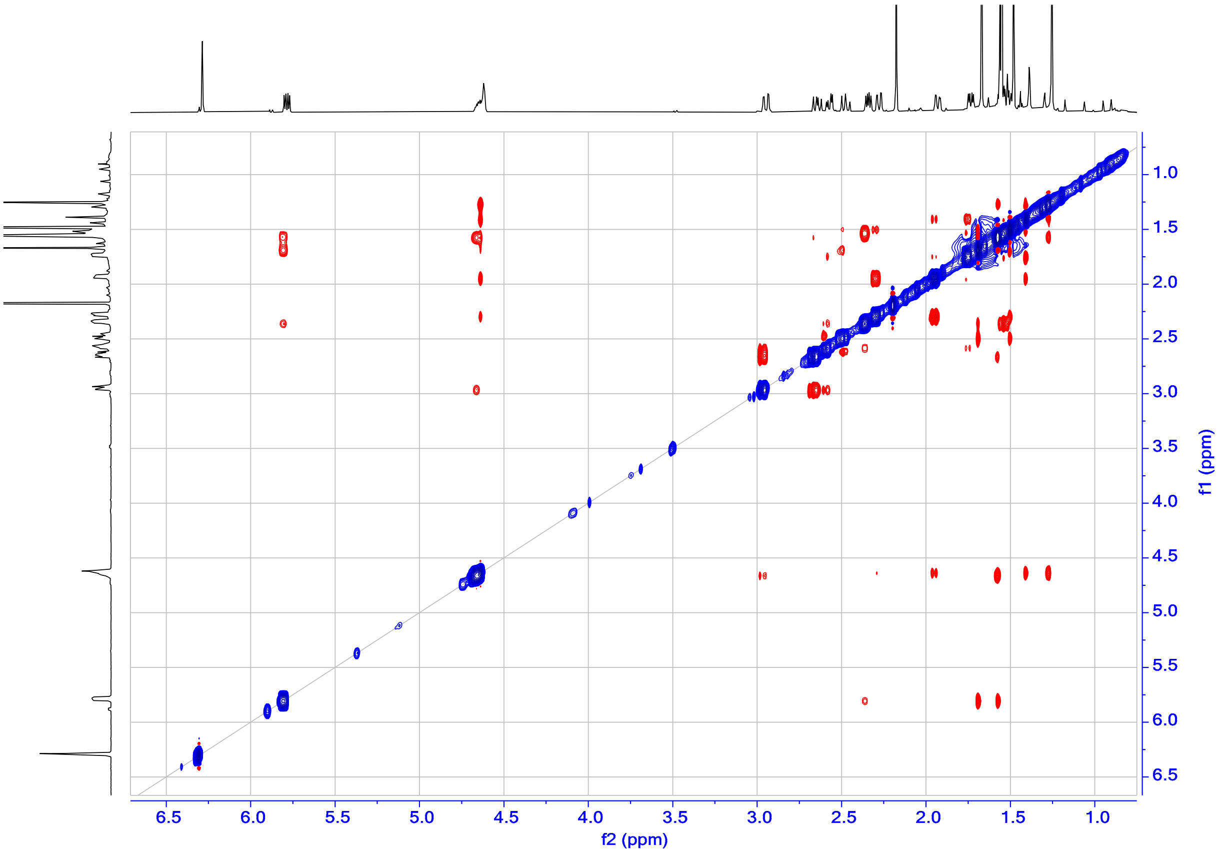

Figure S24. NOESY spectrum of **3**

Table S1. Conformational analysis of the B3LYP/6-31G(d) optimized conformers of (5R,6R,8R,9R,10S,8'R)-2 in the gas phase (T=298.15 K)

Conformer	E[a] (Hartree)	C[b] (Hartree)	G[c] (kcal/mol)	ΔG[d] (kcal/mol)	Population[e]
(5R,6R,8R,9R,10S,8'R)-2-1	-1423.186648	0.475166	-892751.455206	0.0	60.35%
(5R,6R,8R,9R,10S,8'R)-2-2	-1423.185868	0.47529	-892750.887551	0.567655	23.14%
(5R,6R,8R,9R,10S,8'R)-2-3	-1423.184098	0.474963	-892749.98193	1.473276	5.01%
(5R,6R,8R,9R,10S,8'R)-2-4	-1423.184315	0.475267	-892749.927833	1.527373	4.57%
(5R,6R,8R,9R,10S,8'R)-2-5	-1423.183267	0.474426	-892749.797797	1.657409	3.67%
(5R,6R,8R,9R,10S,8'R)-2-6	-1423.184594	0.475868	-892749.725465	1.729741	3.25%

[a]Electronic energy obtained at M062X/6-311+G(2d,p) level of theory; [b]Thermal correction to Gibbs free energy obtained at B3LYP/6-31G(d) level of theory; [c]Gibbs free energy (E + C); [d]The relative Gibbs free energy; [e]The Boltzmann distribution of each conformer.

Table S2. Atomic coordinates (Å) of (5R,6R,8R,9R,10S,8'R)-2-1 obtained at the B3LYP/6-31G(d) level of theory in the gas phase.

C	-2.154062	1.956113	-0.316756	H	-0.795514	-0.128771	-1.167350
C	-3.521333	2.655999	-0.291806	H	-2.781569	-0.163445	2.320292

C	-4.616698	1.795588	-0.895658	H	-1.346114	0.871464	2.340297
C	-4.742617	0.361662	-0.348236	H	-2.929344	1.575112	2.110999
C	-3.305615	-0.271624	-0.354324	H	0.496115	1.561156	-0.088964
C	-3.279285	-1.774946	-0.017745	H	0.424246	0.776899	1.470674
C	-1.912429	-2.389003	-0.334948	H	5.862192	-1.956016	-0.752226
C	-0.722995	-1.647199	0.275332	H	2.612218	-3.341880	-0.994427
C	-0.815539	-0.147094	-0.067869	H	2.346837	2.416729	0.364889
C	-2.161737	0.548763	0.331510	H	3.339354	1.790686	1.676134
C	0.456575	0.578720	0.392251	H	4.271530	2.424592	-1.162201
C	-0.514405	-1.987395	1.752782	H	5.733511	4.099608	-0.030247
C	-5.672652	-0.436236	-1.277416	H	4.017897	4.458719	0.268607
C	-5.432137	0.457939	1.035405	H	5.003862	3.691537	1.535892
C	-2.315740	0.706398	1.863302	H	-5.955715	-1.390647	-0.824110
C	1.705977	-0.202744	0.049211	H	-5.201865	-0.634193	-2.246917
C	2.979023	0.361474	0.110979	H	-6.581537	0.137220	-1.469176
C	4.130007	-0.378746	-0.235269	H	-5.492009	-0.531643	1.489988
C	3.997168	-1.736746	-0.635767	H	-4.909710	1.112440	1.733394
C	2.727046	-2.304828	-0.703123	H	-6.445906	0.851318	0.901561
C	1.607687	-1.553133	-0.357459	H	-4.008902	-2.274107	-0.667825
C	3.206399	1.776231	0.584115	H	-3.650259	-2.942566	1.507547
C	4.440347	2.373027	-0.077740	H	-1.891762	-3.442883	-0.026304
C	4.822856	3.740555	0.457139	H	-1.778980	-2.384622	-1.422867
C	5.469919	0.200576	-0.130931	H	-1.405623	-1.769886	2.339205
O	-3.664306	-1.986014	1.347894	H	-0.297712	-3.057420	1.833368
O	5.054578	-2.502542	-0.939492	H	0.334625	-1.443414	2.175648
O	0.415784	-2.196306	-0.451252	H	-1.422276	2.597158	0.187200
O	5.592039	1.520011	0.131767	H	-1.827448	1.866720	-1.362029
O	6.504595	-0.454696	-0.252543	H	-3.490554	3.598686	-0.844689
O	-5.358909	2.231426	-1.756088	H	-3.802179	2.896673	0.741472
H	-3.032233	-0.260368	-1.421992	-	-	-	-

Table S3. Atomic coordinates (Å) of (5R,6R,8R,9R,10S,8'R)-2-2 obtained at the B3LYP/6-31G(d) level of theory in the gas phase.

C	2.237746	-1.900520	-0.625751	H	0.823016	0.232621	-1.218443
C	3.630212	-2.549043	-0.648815	H	2.935640	-1.836769	1.860685
C	4.707327	-1.573039	-1.085511	H	2.676263	-0.158068	2.313211
C	4.761724	-0.231457	-0.330543	H	1.306059	-1.263012	2.121500
C	3.301982	0.346384	-0.296946	H	-0.440687	-1.637535	-0.421905
C	3.205760	1.782103	0.252312	H	-0.440750	-1.061536	1.230843
C	1.828888	2.390266	-0.029459	H	-5.851792	1.683951	-1.180738
C	0.647299	1.530051	0.419759	H	-2.691127	3.254188	-0.851045
C	0.809270	0.097100	-0.127212	H	-3.036857	-2.565380	-0.915231
C	2.168496	-0.602897	0.217990	H	-2.363538	-2.444202	0.707357
C	-0.447498	-0.726959	0.185279	H	-4.706358	-3.416514	0.575536
C	0.373847	1.647145	1.920933	H	-5.674767	-2.080226	2.442827
C	5.688196	0.725577	-1.099192	H	-3.960303	-2.446242	2.735350
C	5.413687	-0.507706	1.047523	H	-4.442327	-0.823902	2.198505
C	2.279721	-0.975769	1.715708	H	5.924438	1.609446	-0.499599
C	-1.711349	0.055832	-0.096389	H	5.238264	1.051999	-2.043481
C	-2.959770	-0.556416	-0.167761	H	6.621955	0.215769	-1.343933
C	-4.122650	0.175955	-0.485170	H	5.420921	0.403710	1.646912

C	-4.020474	1.565675	-0.766599	H	4.901295	-1.279470	1.622669
C	-2.779196	2.190858	-0.663469	H	6.446435	-0.838769	0.891405
C	-1.650341	1.448849	-0.326894	H	3.938812	2.394304	-0.287744
C	-3.135109	-2.040027	0.045265	H	3.473197	2.727383	1.943723
C	-4.505466	-2.344494	0.645814	H	1.757603	3.387145	0.425803
C	-4.655339	-1.892145	2.094931	H	1.734595	2.538309	-1.111284
C	-5.433077	-0.471153	-0.562104	H	0.128767	2.689542	2.148168
O	3.532161	1.805179	1.649169	H	-0.477560	1.031934	2.224631
O	-5.081531	2.307298	-1.114355	H	1.246734	1.361168	2.505181
O	-0.484729	2.141875	-0.266354	H	1.515434	-2.633023	-0.248452
O	-5.557730	-1.754595	-0.159875	H	1.943314	-1.672276	-1.659303
O	-6.440746	0.090817	-0.991014	H	3.654110	-3.404388	-1.329295
O	5.488086	-1.851249	-1.976565	H	3.887362	-2.922661	0.350295
H	3.064459	0.482878	-1.364389	-	-	-	-

Table S4. Atomic coordinates (Å) of (5R,6R,8R,9R,10S,8'R)-2-3 obtained at the B3LYP/6-31G(d) level of theory in the gas phase.

C	-2.145122	1.953346	-0.280542	H	-0.785713	-0.139654	-1.122942
C	-3.512096	2.651992	-0.255750	H	-3.048641	1.487677	2.128795
C	-4.582876	1.807891	-0.913971	H	-2.664585	-0.214800	2.360041
C	-4.726975	0.353376	-0.415682	H	-1.383132	1.006558	2.345478
C	-3.293365	-0.285261	-0.345537	H	0.492528	1.556683	-0.017348
C	-3.277387	-1.783515	0.042144	H	0.428949	0.740610	1.527451
C	-1.919358	-2.410920	-0.263746	H	5.845573	-1.940272	-0.832863
C	-0.722289	-1.658317	0.321353	H	2.594887	-3.331109	-1.031685
C	-0.815097	-0.157179	-0.023614	H	2.344130	2.405214	0.426425
C	-2.163301	0.542901	0.357964	H	3.363228	1.757508	1.706503
C	0.455800	0.565048	0.444822	H	4.238987	2.443887	-1.137428
C	-0.480979	-2.002871	1.792934	H	4.009065	4.452030	0.333492
C	-5.598338	-0.407729	-1.430149	H	5.020093	3.664733	1.568247
C	-5.514235	0.423118	0.918673	H	5.719400	4.101812	-0.003839
C	-2.327940	0.703498	1.885824	H	-5.089861	-0.508738	-2.394446
C	1.702017	-0.209819	0.075348	H	-6.526392	0.139248	-1.607908
C	2.974723	0.355736	0.123909	H	-5.853141	-1.407580	-1.066961
C	4.120799	-0.376350	-0.255193	H	-4.932737	0.812884	1.753644
C	3.982681	-1.727981	-0.675405	H	-6.395380	1.061100	0.788412
C	2.712968	-2.298249	-0.727145	H	-5.901057	-0.564674	1.197492
C	1.598080	-1.555244	-0.348930	H	-4.011348	-2.305728	-0.584763
C	3.208786	1.762465	0.617240	H	-4.478254	-1.752608	1.593387
C	4.428713	2.373437	-0.057437	H	-1.915927	-3.448738	0.084359
C	4.818694	3.732281	0.494104	H	-1.791247	-2.432113	-1.351424
C	5.460904	0.204212	-0.167662	H	-1.368073	-1.818225	2.395990
O	-3.575350	-2.051923	1.417550	H	-0.243524	-3.068884	1.858795
O	5.035745	-2.485805	-1.012782	H	0.363513	-1.445167	2.206888
O	0.407177	-2.199855	-0.429404	H	-1.416698	2.589597	0.233811
O	5.585655	1.519780	0.115114	H	-1.812179	1.873173	-1.324473
O	6.494786	-0.445641	-0.321287	H	-3.475867	3.614695	-0.772517
O	-5.302195	2.261364	-1.784033	H	-3.816011	2.850930	0.780384
H	-2.985971	-0.298597	-1.403569	-	-	-	-

Table S5. Atomic coordinates (Å) of (5R,6R,8R,9R,10S,8'R)-2-4 obtained at the B3LYP/6-31G(d) level of

theory in the gas phase.

C	-2.093668	1.892537	-0.666781	H	-0.709587	-0.209720	-1.223798
C	-3.437490	2.333046	-1.289889	H	-1.543337	1.431967	2.035827
C	-4.616045	1.844858	-0.476532	H	-3.258890	1.585577	1.704179
C	-4.741490	0.315197	-0.366262	H	-2.620645	0.030430	2.224849
C	-3.309631	-0.327616	-0.456592	H	0.475242	1.552079	-0.051722
C	-3.259234	-1.803928	-0.018779	H	0.353623	0.713584	1.477870
C	-1.900336	-2.427579	-0.349501	H	5.882984	-1.932653	-0.656110
C	-0.722157	-1.669624	0.265673	H	2.651400	-3.355631	-0.920146
C	-0.804141	-0.182594	-0.128296	H	2.310831	2.417465	0.366684
C	-2.159614	0.550082	0.134577	H	3.299989	1.817199	1.692010
C	0.434537	0.553391	0.395473	H	4.247979	2.423446	-1.147182
C	-0.532151	-1.958748	1.756625	H	5.681942	4.130505	-0.027132
C	-5.557049	-0.118243	-1.613531	H	3.959875	4.475301	0.250939
C	-5.576925	-0.032904	0.877088	H	4.942332	3.737767	1.538280
C	-2.405761	0.905906	1.614839	H	-6.554194	0.331810	-1.589410
C	1.699459	-0.212211	0.072728	H	-5.678915	-1.206757	-1.627244
C	2.965905	0.365649	0.141419	H	-5.069741	0.176569	-2.549774
C	4.128551	-0.366812	-0.182594	H	-5.049480	0.181221	1.806408
C	4.014644	-1.731386	-0.565733	H	-6.490145	0.566572	0.858462
C	2.751367	-2.313557	-0.641498	H	-5.842079	-1.091498	0.884899
C	1.619630	-1.570237	-0.319329	H	-4.027891	-2.345096	-0.591817
C	3.175105	1.788325	0.599404	H	-3.496671	-2.866667	1.602578
C	4.407859	2.389543	-0.060625	H	-1.876516	-3.477372	-0.027778
C	4.770755	3.768820	0.457356	H	-1.764405	-2.433225	-1.437125
C	5.461181	0.227954	-0.072119	H	-0.420254	-3.039065	1.895330
O	-3.551526	-1.925122	1.377049	H	0.374937	-1.481921	2.137227
O	5.083442	-2.490220	-0.846075	H	-1.385535	-1.619260	2.340226
O	0.436954	-2.231489	-0.419205	H	-1.737532	2.681834	0.002759
O	5.567108	1.552381	0.170890	H	-1.356777	1.814854	-1.473462
O	6.503827	-0.418579	-0.171441	H	-3.525620	1.901220	-2.295303
O	-5.423560	2.605278	0.023500	H	-3.498993	3.419070	-1.389200
H	-3.083692	-0.386803	-1.531775	-	-	-	-

Table S6. Atomic coordinates (Å) of (5R,6R,8R,9R,10S,8'R)-2-5 obtained at the B3LYP/6-31G(d) level of theory in the gas phase.

C	-2.235340	1.675268	-1.092475	H	-0.721155	-0.365418	-1.275325
C	-3.588943	2.403001	-1.150961	H	-2.344369	0.427412	2.161160
C	-4.762927	1.668525	-0.525601	H	-1.601833	1.932559	1.579551
C	-4.744453	0.129542	-0.445524	H	-3.335770	1.689099	1.449035
C	-3.284624	-0.461059	-0.398643	H	0.439399	1.613388	-0.469568
C	-3.163658	-1.818240	0.333393	H	0.375152	1.051725	1.188835
C	-1.797963	-2.446554	0.063587	H	5.888341	-1.661642	-1.080001
C	-0.625981	-1.541401	0.450943	H	2.747089	-3.268260	-0.738980
C	-0.788819	-0.149087	-0.199012	H	3.021045	2.555841	-0.948666
C	-2.170222	0.558863	-0.020138	H	2.324194	2.462613	0.666331
C	0.434503	0.709374	0.147308	H	4.656838	3.460186	0.548895
C	-0.339975	-1.562482	1.954947	H	5.614732	2.173829	2.456634
C	-5.412845	-0.351497	-1.759645	H	3.891921	2.524458	2.717559
C	-5.665675	-0.270383	0.722413	H	4.400857	0.897874	2.220593
C	-2.369423	1.179075	1.374507	H	-6.441731	0.014773	-1.828178

C	1.715522	-0.063271	-0.083985	H	-5.432356	-1.447019	-1.791707
C	2.956304	0.562914	-0.155479	H	-4.862291	-0.002113	-2.640454
C	4.132680	-0.161559	-0.440580	H	-6.640235	0.200702	0.587780
C	4.051330	-1.558999	-0.688631	H	-5.820896	-1.354470	0.755063
C	2.817935	-2.198827	-0.581188	H	-5.282035	0.084687	1.684873
C	1.674931	-1.465174	-0.274516	H	-3.926454	-2.494818	-0.083522
C	3.110361	2.053253	0.024869	H	-4.226517	-1.510646	1.949295
C	4.468154	2.387319	0.638309	H	-1.724942	-3.399989	0.595895
C	4.602697	1.966239	2.098345	H	-1.713984	-2.660131	-1.007514
C	5.435050	0.500536	-0.516719	H	-1.199783	-1.224039	2.529101
O	-3.313694	-1.758715	1.753254	H	-0.123479	-2.593808	2.249169
O	5.126216	-2.294094	-1.007614	H	0.532424	-0.953386	2.205504
O	0.519970	-2.173698	-0.199224	H	-1.449849	2.416124	-0.914191
O	5.538382	1.794343	-0.140839	H	-2.013722	1.220847	-2.065790
O	6.455345	-0.056717	-0.921603	H	-3.863668	2.611210	-2.193904
O	-5.732586	2.298047	-0.141789	H	-3.554239	3.378508	-0.657528
H	-3.053460	-0.725675	-1.440570	-	-	-	-

Table S7. Atomic coordinates (Å) of (5R,6R,8R,9R,10S,8'R)-2-6 obtained at the B3LYP/6-31G(d) level of theory in the gas phase.

C	-2.087056	1.893318	-0.653780	H	-0.708647	-0.216846	-1.213397
C	-3.425203	2.339092	-1.286702	H	-2.604979	0.006263	2.224207
C	-4.606454	1.848113	-0.483628	H	-1.557059	1.427769	2.045908
C	-4.733236	0.316281	-0.390548	H	-3.274225	1.559098	1.716626
C	-3.302761	-0.332751	-0.458677	H	0.474685	1.550459	-0.042323
C	-3.257063	-1.812074	-0.000332	H	0.355105	0.710286	1.487092
C	-1.902015	-2.438145	-0.322814	H	5.879472	-1.930204	-0.672655
C	-0.721576	-1.673379	0.281193	H	2.647478	-3.353734	-0.928279
C	-0.803632	-0.186988	-0.117909	H	2.310109	2.415223	0.379159
C	-2.158786	0.546573	0.139465	H	3.304473	1.810204	1.698529
C	0.434338	0.551373	0.404284	H	4.241499	2.426797	-1.141963
C	-0.516936	-1.963786	1.769476	H	4.945208	3.732402	1.545306
C	-5.533610	-0.109749	-1.649590	H	5.679211	4.130555	-0.021328
C	-5.596321	-0.027252	0.836486	H	3.957989	4.473912	0.263890
C	-2.411206	0.891745	1.621135	H	-6.536005	0.329417	-1.632568
C	1.698668	-0.213620	0.077534	H	-5.640621	-1.199564	-1.680218
C	2.964851	0.364291	0.143879	H	-5.037922	0.202359	-2.574863
C	4.126810	-0.366639	-0.186905	H	-5.074340	0.143646	1.780834
C	4.011381	-1.729943	-0.574583	H	-6.476092	0.619492	0.838375
C	2.748220	-2.312343	-0.647435	H	-5.955304	-1.062875	0.791351
C	1.616865	-1.571058	-0.318214	H	-4.021082	-2.361910	-0.571342
C	3.175390	1.785331	0.606300	H	-4.386785	-1.776287	1.601580
C	4.405594	2.389158	-0.056093	H	-1.892088	-3.476613	0.022441
C	4.769801	3.766945	0.465091	H	-1.767430	-2.451101	-1.409988
C	5.459244	0.227886	-0.079400	H	0.380386	-1.467396	2.148645
O	-3.477085	-2.027350	1.395888	H	-1.378451	-1.656763	2.358116
O	5.079588	-2.487239	-0.862088	H	-0.384131	-3.041828	1.899614
O	0.434886	-2.231181	-0.415705	H	-1.735996	2.679647	0.021872
O	5.565731	1.551975	0.168444	H	-1.344832	1.819309	-1.455496
O	6.502253	-0.416964	-0.186126	H	-3.505195	1.911437	-2.294359
O	-5.414315	2.601451	0.026960	H	-3.485208	3.425562	-1.381196

H	-3.069529	-0.398531	-1.531964	-	-	-	-

Table S8. Key transitions, oscillator strengths, and rotatory strengths in the ECD spectrum of conformer (5R,6R,8R,9R,10S,8'R)-2-1 at the CAM-B3LYP/6-311G(d) level of theory in MeOH with IEFPCM solvent model.

Num[a]	*Transition*[b]	*CI-coeff*[b]	*ΔE (eV)*[d]	*λ (nm)*[e]	*f*[f]	R_{vel}[g]	R_{len}[h]
1	113->117	0.67683	4.3812	282.99	0.0003	-3.1984	-3.4897
2	115->116	0.6566	4.4596	278.02	0.1563	9.8347	9.9402
3	114->116	0.63011	4.9997	247.98	0.2759	-6.242	-2.7916
4	110->116	0.62571	5.5399	223.80	0.0014	0.3847	0.7249
5	114->116	-0.253	5.8917	210.44	0.3439	0.5517	0.9708
	114->118	0.25002					
	115->118	0.58338					
6	114->118	0.60609	6.2472	198.46	0.6294	27.7451	28.9367
	115->118	-0.28124					
7	108->116	0.48541	6.6312	186.97	0.0809	27.3005	27.8573
	111->116	-0.36205					
8	108->116	0.41326	6.8397	181.27	0.0806	-3.2569	-4.221
	111->116	0.31014					
	113->116	-0.31613					
9	107->116	-0.36415	7.0401	176.11	0.0382	-12.247	-15.1454
	113->116	0.49024					
10	107->116	0.2496	7.1078	174.44	0.0212	5.6886	4.2361
	112->116	0.23566					
	113->116	0.35872					
11	115->117	0.60634	7.1465	173.49	0.0042	-4.3795	-4.1686
12	112->116	0.51175	7.3084	169.65	0.0183	-10.2122	-10.5469
13	109->117	-0.33894	7.3286	169.18	0.0047	-2.3884	-3.4148
	112->117	0.51147					
14	115->119	0.27547	7.3887	167.80	0.0672	-8.5243	-9.1228
	115->120	0.32535					
15	111->117	0.46905	7.4369	166.71	0.0084	3.1827	4.0586
	114->117	0.24976					
16	112->119	0.46598	7.5068	165.16	0.0027	-12.7741	-15.4998
17	103->116	0.4016	7.5283	164.69	0.0181	-13.2369	-15.408
18	115->120	0.33185	7.5781	163.61	0.0319	-10.4629	-10.1689
	115->121	-0.31233					
	115->123	-0.31643					
19	114->117	0.56804	7.6691	161.67	0.0010	3.4803	3.2157
20	110->118	0.42985	7.6786	161.47	0.0038	-8.9404	-7.9278
21	113->119	0.32599	7.7736	159.49	0.0216	20.5351	22.0926
22	113->119	0.45588	7.7984	158.99	0.0308	-23.0458	-20.7142
23	115->119	0.27921	7.8129	158.69	0.0051	-15.3707	-13.9016
	115->120	-0.22917					
24	114->120	0.25266	7.9092	156.76	0.0542	-2.978	-3.657
	114->121	0.29846					
	114->123	0.25848					
	115->119	-0.2415					
25	110->118	-0.24152	7.9420	156.11	0.0745	-6.4222	-8.2304
	111->118	0.42676					
26	105->117	0.27253	7.9898	155.18	0.0242	3.9923	4.9202

	106->117	0.34377					
	109->117	-0.24109					
	112->117	-0.29728					
27	115->122	0.31256	8.0430	154.15	0.0028	-6.5814	-5.6446
	115->123	0.23824					
	115->124	0.34652					
28	109->116	0.3125	8.0758	153.53	0.0033	13.5829	12.5718
29	109->116	0.35732	8.0861	153.33	0.0227	-36.5946	-36.4917
30	106->117	0.28389	8.0995	153.08	0.0361	7.2921	6.0946
	109->117	0.3681					
	112->117	0.31062					
31	104->116	0.27811	8.1483	152.16	0.0112	11.8763	11.9418
	114->120	0.39439					
32	104->116	0.34548	8.1659	151.83	0.0111	13.7143	12.6226
	106->116	0.29038					
33	107->118	-0.2373	8.1881	151.42	0.0739	21.7687	20.349
	108->118	-0.32482					
	113->118	0.42482					
34	108->118	0.26459	8.3021	149.34	0.0104	31.5523	34.5882
	113->118	0.38677					
35	113->120	-0.26131	8.3282	148.87	0.0465	-12.7483	-11.0227
	113->121	0.30248					
36	114->119	-0.2487	8.3662	148.20	0.0011	-4.1912	-7.5896
	114->120	0.25584					
	115->126	0.24061					

[a]Number of the excited states; [b]Only transitions with contribution over 10.0% were listed; [c]Configuration-interaction coefficient; [d]Excitation energy; [e]Wavelength; [f]Oscillator strength; [g]Rotatory strength in velocity form (10^{-40} cgs); [h]Rotatory strength in length form (10^{-40} cgs).

Table S9. Key transitions, oscillator strengths, and rotatory strengths in the ECD spectrum of conformer (5R,6R,8R,9R,10S,8'R)-2-2 at the CAM-B3LYP/6-311G(d) level of theory in MeOH with IEFPCM solvent model.

Num[a]	***Transition***[b]	***CI-coeff***[b]	***ΔE (eV)***[d]	***λ (nm)***[e]	***f***[f]	R_{vel}[g]	R_{len}[h]
1	113->117	0.67723	4.3826	282.90	0.0003	-3.0472	-3.1379
2	115->116	0.65654	4.4539	278.37	0.1540	-8.4546	-8.901
3	114->116	0.63389	4.9874	248.60	0.2712	42.2203	42.4225
4	110->116	0.60491	5.5234	224.47	0.0009	0.264	0.5465
5	114->116	-0.24573	5.8901	210.50	0.3240	-6.3201	-6.3226
	114->118	0.25451					
	115->118	0.58501					
6	114->118	0.60352	6.2328	198.92	0.6249	37.6144	41.226
	115->118	-0.28804					
7	108->116	0.42284	6.6769	185.69	0.0901	24.5931	24.4748
	109->116	0.22452					
	111->116	0.38717					
8	103->116	-0.24203	6.8409	181.24	0.0614	-2.9087	-4.0331
	105->116	0.23648					
	108->116	0.34125					
	111->116	-0.23089					
	113->116	0.2738					
9	107->116	0.32308	7.0345	176.25	0.0182	-8.6356	-7.0366

	113->116	0.55714					
10	107->116	-0.27908	7.1381	173.69	0.0156	15.8915	16.903
	115->117	0.49897					
11	107->116	0.38576	7.1433	173.57	0.0358	-23.7307	-18.3017
	115->117	0.38256					
12	112->116	0.52467	7.3114	169.58	0.0025	-2.7769	-2.4167
13	109->117	-0.29632	7.3295	169.16	0.0157	4.5522	3.5133
	112->117	0.48402					
14	115->117	-0.24843	7.3790	168.02	0.0086	-4.1156	-3.962
	115->119	0.26775					
	115->120	0.46031					
15	103->116	0.45643	7.4109	167.30	0.0135	-17.6357	-16.386
16	111->117	0.45787	7.4338	166.78	0.0153	13.41	14.2043
	114->117	-0.28415					
17	115->120	-0.23226	7.4834	165.68	0.0738	1.9261	0.8197
	115->122	0.42056					
	115->123	-0.24311					
18	112->119	0.49388	7.5084	165.13	0.0133	-32.956	-36.4404
19	114->117	0.57775	7.6555	161.95	0.0017	6.6462	6.47
20	110->118	0.46302	7.6753	161.54	0.0007	-5.9347	-4.446
21	114->119	0.25726	7.7730	159.51	0.0248	-1.366	1.4795
	115->119	0.36467					
	115->120	-0.22538					
	115->122	-0.23953					
22	113->119	0.36463	7.7846	159.27	0.0004	-3.3072	-2.0205
23	113->119	0.37779	7.7980	158.99	0.0103	-3.8092	-3.6229
24	111->118	-0.29616	7.9125	156.69	0.1562	-8.6804	-12.5975
	114->122	0.35164					
	114->123	-0.2324					
25	111->118	0.29381	7.9372	156.21	0.0165	-16.0736	-14.6091
	114->119	0.28516					
	114->120	0.28267					
26	111->118	-0.24359	7.9696	155.57	0.0107	13.8592	14.465
	114->120	0.3819					
	114->122	-0.23656					
27	105->117	-0.26094	7.9951	155.08	0.0176	10.3935	10.9528
	106->117	0.32055					
	112->117	0.26859					
28	115->121	0.28094	8.0814	153.42	0.0130	-25.0486	-24.7568
	115->125	0.3225					
29	109->116	0.42604	8.0931	153.20	0.0109	14.3741	12.8303
30	106->117	-0.27891	8.1041	152.99	0.0314	-7.2236	-8.2314
	109->117	0.34291					
	112->117	0.30056					
31	114->120	0.27701	8.1243	152.61	0.0204	8.5051	8.9931
	114->122	0.23995					
	115->121	0.2664					
	115->125	0.23502					
32	104->116	0.431	8.1595	151.95	0.0093	3.6978	2.148
	106->116	0.33486					
33	107->118	0.25774	8.1887	151.41	0.0607	4.0307	5.7841

	108->118	0.3039					
	113->118	0.40515					
34	113->118	0.24081	8.3006	149.37	0.0075	37.3064	36.9968
35	113->118	0.38647	8.3154	149.10	0.0124	10.3303	9.9258
36	113->121	0.24151	8.3420	148.63	0.0240	-49.2064	-45.9173
	113->123	0.28063					

[a]Number of the excited states; [b]Only transitions with contribution over 10.0% were listed; [c]Configuration-interaction coefficient; [d]Excitation energy; [e]Wavelength; [f]Oscillator strength; [g]Rotatory strength in velocity form (10^{-40} cgs); [h]Rotatory strength in length form (10^{-40} cgs).

Table S10. Key transitions, oscillator strengths, and rotatory strengths in the ECD spectrum of conformer (5R,6R,8R,9R,10S,8'R)-2-3 at the CAM-B3LYP/6-311G(d) level of theory in MeOH with IEFPCM solvent model.

Num[a]	***Transition***[b]	***CI-coeff***[b]	***ΔE (eV)***[d]	***λ (nm)***[e]	***f***[f]	R_{vel}[g]	R_{len}[h]
1	112->117	0.23477	4.3767	283.28	0.0003	-1.0274	-2.0466
	113->117	0.64713					
2	115->116	0.65558	4.4600	277.99	0.1570	10.0159	10.114
3	114->116	0.62962	4.9942	248.26	0.2772	-5.5157	-1.7498
4	110->116	0.4083	5.5398	223.81	0.0014	0.4081	0.7377
	111->116	0.45847					
5	114->116	-0.25299	5.8885	210.55	0.3393	-0.7414	-0.2212
	114->118	0.25045					
	115->118	0.5832					
6	114->118	0.60644	6.2472	198.46	0.6318	31.0014	32.3656
	115->118	-0.28122					
7	108->116	0.29938	6.6218	187.24	0.0751	26.3831	26.8263
	109->116	0.34355					
	110->116	-0.32573					
8	108->116	0.47051	6.8184	181.84	0.0813	-2.5405	-3.2287
	113->116	-0.36971					
9	107->116	-0.36391	7.0266	176.45	0.0442	-12.973	-16.3148
	113->116	0.43459					
10	112->117	0.28322	7.0588	175.64	0.0023	0.1282	0.0513
	115->117	0.57741					
11	103->116	0.29061	7.1396	173.66	0.0279	0.3245	-0.8173
	107->116	0.3948					
	113->116	0.32133					
12	112->117	0.49653	7.1776	172.74	0.0068	-11.1806	-11.0495
	115->117	-0.32473					
13	115->119	0.33294	7.3600	168.46	0.0436	0.6789	-0.3243
	115->120	0.3831					
14	109->117	-0.32155	7.4232	167.02	0.0338	-7.7461	-8.5109
	111->117	0.41552					
	114->117	-0.30617					
15	112->116	0.43607	7.4477	166.47	0.0038	16.2041	16.9482
16	112->119	-0.28263	7.4844	165.66	0.0100	-10.5574	-6.2725
	113->119	0.47301					
17	103->116	0.39222	7.5263	164.73	0.0159	-3.3445	-4.2864
	112->116	-0.23764					
18	114->117	-0.24517	7.5663	163.86	0.0353	-8.9357	-7.6298
	115->120	0.28652					

	115->122	0.32998					
	115->124	0.24519					
19	114->117	0.54044	7.5993	163.15	0.0012	2.6963	2.6854
20	110->118	0.39117	7.7118	160.77	0.0030	1.4243	0.9607
	111->118	0.27289					
21	114->119	0.29098	7.7829	159.30	0.0623	14.0035	13.164
22	99->116	-0.22851	7.8323	158.30	0.0049	-22.9126	-22.3819
	115->119	0.23046					
23	112->119	0.3287	7.8947	157.05	0.0027	7.3296	11.3287
	113->119	0.30323					
24	114->122	0.22536	7.9140	156.66	0.0170	1.0714	0.5841
25	99->116	0.22781	7.9368	156.21	0.1030	-15.5186	-16.8389
	111->118	0.28297					
26	109->116	0.38893	7.9556	155.85	0.0025	5.3134	4.9764
	111->118	-0.24679					
	112->116	0.24829					
27	105->117	0.5055	7.9987	155.01	0.0259	-6.0743	-5.7623
28	115->123	0.49534	8.0479	154.06	0.0025	-2.8606	-3.091
	115->124	-0.32012					
29	114->119	0.25922	8.0928	153.20	0.0201	1.1643	2.4516
	114->122	0.24948					
	114->124	0.26644					
	114->126	-0.23104					
30	104->116	-0.23044	8.1354	152.40	0.0037	-9.8187	-10.79
	106->116	0.49406					
31	114->120	0.40963	8.1458	152.21	0.0105	21.1898	20.4343
32	107->118	-0.26507	8.1681	151.79	0.0906	11.7	9.5415
	113->118	0.38948					
33	98->117	-0.26966	8.2824	149.70	0.0226	-3.518	-4.8697
	106->117	0.32456					
	113->121	0.24876					
34	107->118	0.25599	8.3166	149.08	0.0332	4.039	4.9595
	108->118	0.34655					
	113->118	0.32806					
35	114->120	0.24163	8.3664	148.19	0.0021	6.2731	6.4057
36	113->121	0.37102	8.3790	147.97	0.0290	16.9196	20.6899
	113->122	-0.31799					

[a]Number of the excited states; [b]Only transitions with contribution over 10.0% were listed; [c]Configuration-interaction coefficient; [d]Excitation energy; [e]Wavelength; [f]Oscillator strength; [g]Rotatory strength in velocity form (10^{-40} cgs); [h]Rotatory strength in length form (10^{-40} cgs).

Table S11. Key transitions, oscillator strengths, and rotatory strengths in the ECD spectrum of conformer (5R,6R,8R,9R,10S,8'R)-2-4 at the CAM-B3LYP/6-311G(d) level of theory in MeOH with IEFPCM solvent model.

Num[a]	***Transition***[b]	***CI-coeff***[b]	***ΔE (eV)***[d]	***λ (nm)***[e]	***f***[f]	R_{vel}[g]	R_{len}[h]
1	113->117	0.67257	4.4018	281.67	0.0004	15.5444	12.839
2	115->116	0.65528	4.4647	277.70	0.1563	9.4705	9.6724
3	114->116	0.62935	4.9971	248.11	0.2776	-7.9709	-4.6538
	115->118	0.22039					
4	110->116	0.62151	5.5405	223.78	0.0014	0.5937	0.9022
5	114->116	-0.25274	5.8968	210.26	0.3413	3.8145	4.1011

	114->118	0.25142					
	115->118	0.58245					
6	114->118	0.6054	6.2526	198.29	0.6318	14.481	15.6187
	115->118	-0.28258					
7	108->116	0.47152	6.6360	186.83	0.0848	25.2163	25.7486
	111->116	-0.36205					
8	108->116	0.38921	6.8467	181.09	0.0760	-2.7131	-3.632
	111->116	0.32388					
	113->116	-0.33581					
9	107->116	-0.28285	7.0036	177.03	0.0325	-6.6679	-8.9417
	113->116	0.50812					
10	115->117	0.49339	7.0931	174.80	0.0146	6.6377	4.6
	115->120	-0.20281					
11	105->116	0.21325	7.1341	173.79	0.0213	-10.5001	-10.0843
	106->116	-0.26068					
	107->116	-0.21348					
	113->116	-0.22642					
	115->117	0.37138					
12	112->116	0.56051	7.3088	169.64	0.0152	-7.2674	-7.8083
13	109->117	0.23712	7.3786	168.03	0.0338	-8.2852	-6.0637
	111->117	-0.34841					
	112->117	0.41223					
	114->117	-0.27091					
14	115->119	0.25484	7.4012	167.52	0.0324	-7.8657	-8.806
	115->120	0.32492					
	115->122	-0.24772					
15	111->117	0.31229	7.4555	166.30	0.0220	5.9837	6.2659
	112->117	0.31308					
16	112->119	0.47576	7.4889	165.56	0.0021	1.5401	-2.3456
	112->121	-0.2527					
17	101->116	0.4218	7.5292	164.67	0.0205	-9.1329	-11.6598
	111->116	-0.2038					
18	115->120	0.38721	7.6162	162.79	0.0110	0.1919	0.7453
	115->122	0.37404					
19	114->117	0.46754	7.6486	162.10	0.0024	-0.3859	0.1438
	114->119	-0.2007					
20	110->118	0.38301	7.6592	161.88	0.0077	-14.9743	-14.5492
21	113->119	0.53821	7.6714	161.62	0.0050	9.3876	8.6378
	113->121	0.29887					
22	99->116	-0.21092	7.7860	159.24	0.0309	9.391	10.4577
	110->118	0.2663					
23	107->117	-0.25253	7.8098	158.75	0.0296	-28.8947	-31.0303
	115->119	0.30282					
	115->120	-0.2045					
24	106->117	-0.31058	7.8145	158.66	0.0050	-8.9732	-7.7086
	107->117	0.34898					
	115->119	0.21478					
25	114->120	-0.26337	7.9020	156.90	0.0617	5.6402	5.2968
	114->122	0.39912					
	115->119	0.22844					
26	110->118	-0.24962	7.9552	155.85	0.0777	-14.4172	-16.2645

	111->118	0.43523					
27	109->116	0.43136	8.0429	154.15	0.0052	-20.2535	-21.355
28	109->117	0.28091	8.0603	153.82	0.0056	-0.6669	0.3362
	112->117	-0.24367					
	114->119	0.20047					
	115->123	0.2005					
29	109->117	0.33153	8.0687	153.66	0.0241	8.2748	7.6353
	112->117	-0.29292					
30	115->121	0.21369	8.0981	153.10	0.0099	-3.7266	-3.9631
	115->123	0.355					
	115->124	-0.29539					
31	104->116	0.42843	8.1232	152.63	0.0014	-4.6132	-5.1389
	105->116	0.21501					
	106->116	0.28443					
32	108->118	-0.2826	8.1594	151.95	0.0294	4.9918	3.8024
	113->118	0.48788					
33	114->120	0.44615	8.1890	151.40	0.0395	30.6903	30.0708
	114->122	0.35273					
34	105->118	-0.24691	8.2878	149.60	0.0465	34.1646	35.7823
	106->118	0.20826					
	108->118	0.37264					
	113->118	0.2817					
35	113->120	0.2114	8.3514	148.46	0.0369	-4.7092	-2.4329
	113->121	0.20972					
36	113->120	0.24274	8.3732	148.07	0.0140	-28.4925	-28.6529

[a]Number of the excited states; [b]Only transitions with contribution over 10.0% were listed; [c]Configuration-interaction coefficient; [d]Excitation energy; [e]Wavelength; [f]Oscillator strength; [g]Rotatory strength in velocity form (10^{-40} cgs); [h]Rotatory strength in length form (10^{-40} cgs).

Table S12. Key transitions, oscillator strengths, and rotatory strengths in the ECD spectrum of conformer (5R,6R,8R,9R,10S,8'R)-2-5 at the CAM-B3LYP/6-311G(d) level of theory in MeOH with IEFPCM solvent model.

Num[a]	***Transition***[b]	***CI-coeff***[b]	***ΔE (eV)***[d]	***λ (nm)***[e]	***f***[f]	R_{vel}[g]	R_{len}[h]
1	112->117	0.23899	4.3930	282.23	0.0005	3.0579	3.784
	113->117	0.64782					
2	115->116	0.65476	4.4567	278.20	0.1544	-8.3557	-8.7507
3	114->116	0.63187	4.9810	248.92	0.2727	41.6867	42.0469
4	109->116	0.23602	5.5247	224.42	0.0009	0.5393	0.8212
	110->116	0.35776					
	111->116	0.49091					
5	114->116	-0.24671	5.8876	210.58	0.3186	-2.6364	-2.5396
	114->118	0.25435					
	115->118	0.58401					
6	114->118	0.60338	6.2348	198.86	0.6255	28.8733	32.8459
	115->118	-0.28723					
7	108->116	-0.28965	6.6599	186.17	0.0724	21.2131	21.2175
	109->116	-0.30832					
	110->116	0.37102					
8	108->116	0.47592	6.8106	182.04	0.0747	-1.6002	-2.952
	113->116	-0.35392					
9	107->116	-0.33724	7.0301	176.36	0.0305	-11.3305	-9.0265

	113->116	0.44412					
10	112->117	0.23764	7.1141	174.28	0.0006	2.5365	2.209
	115->117	0.60693					
11	107->116	0.50997	7.1628	173.09	0.0429	-7.952	-1.7218
	113->116	0.27744					
12	112->117	0.52433	7.2096	171.97	0.0051	1.6774	1.2444
	115->117	-0.27419					
13	115->119	0.3552	7.3513	168.66	0.0062	9.797	10.1586
	115->120	0.5133					
14	103->116	0.31573	7.3977	167.60	0.0124	-8.6113	-8.5069
	111->116	-0.24368					
15	115->122	0.45535	7.4723	165.93	0.0446	-6.6891	-6.332
16	109->117	-0.2303	7.4773	165.81	0.0600	1.1655	1.8513
	110->117	-0.30257					
	111->117	0.40381					
	114->117	-0.28021					
17	112->116	0.41235	7.5196	164.88	0.0061	11.2378	11.6484
18	113->119	0.592	7.5766	163.64	0.0004	1.1078	0.5618
19	114->117	0.58521	7.6114	162.89	0.0058	2.5281	4.3675
20	110->118	0.28547	7.7167	160.67	0.0008	-3.1179	-3.1713
	111->118	0.3959					
	112->116	0.26142					
21	114->119	0.23663	7.7668	159.63	0.0252	-8.8964	-6.8782
	115->119	0.38141					
	115->120	-0.28074					
	115->123	-0.2799					
22	109->116	0.40134	7.8029	158.89	0.0051	6.1914	6.2438
	112->116	0.35664					
23	98->116	0.33657	7.8581	157.78	0.0023	2.0369	0.2297
	99->116	-0.2325					
	111->116	0.23558					
24	109->118	0.27767	7.8869	157.20	0.1021	-13.5453	-11.9969
	113->118	0.24205					
25	105->117	0.33614	7.9068	156.81	0.0192	-15.3301	-15.0851
	106->117	0.30762					
	109->117	0.25873					
26	114->119	-0.26773	7.9192	156.56	0.0618	-22.2317	-26.2667
	114->122	0.42685					
27	112->119	0.3308	7.9233	156.48	0.0168	79.1897	79.8444
28	114->119	0.25311	7.9355	156.24	0.0113	11.4544	11.2734
	114->120	0.43374					
29	115->121	-0.32424	8.0777	153.49	0.0025	-7.1913	-7.7576
	115->125	0.39071					
30	105->116	-0.2523	8.1205	152.68	0.0097	-19.7974	-22.696
	106->116	0.35649					
31	113->118	0.26479	8.1395	152.32	0.0086	3.7687	2.4585
32	110->118	0.24545	8.1690	151.77	0.0880	-13.9489	-11.4043
	113->118	0.30566					
33	100->117	-0.24159	8.1812	151.55	0.0102	-10.3305	-7.9668
	104->117	0.40746					
34	104->117	0.31187	8.2823	149.70	0.0430	-6.8199	-9.354

	105->117	0.2403					
	106->117	0.22637					
	109->117	-0.27243					
35	114->119	0.32157	8.2949	149.47	0.0026	3.7295	2.995
36	107->118	0.31822	8.3183	149.05	0.0271	14.0447	15.3271
	108->118	0.32509					
	113->118	0.31981					

[a]Number of the excited states; [b]Only transitions with contribution over 10.0% were listed; [c]Configuration-interaction coefficient; [d]Excitation energy; [e]Wavelength; [f]Oscillator strength; [g]Rotatory strength in velocity form (10^{-40} cgs); [h]Rotatory strength in length form (10^{-40} cgs).

Table S13. Key transitions, oscillator strengths, and rotatory strengths in the ECD spectrum of conformer (5R,6R,8R,9R,10S,8'R)-2-6 at the CAM-B3LYP/6-311G(d) level of theory in MeOH with IEFPCM solvent model.

Num[a]	*Transition*[b]	*CI-coeff*[b]	*ΔE (eV)*[d]	*λ (nm)*[e]	*f*[f]	R_{vel}[g]	R_{len}[h]
1	112->117	0.2324	4.4122	281.00	0.0004	15.3832	12.4545
	113->117	0.64639					
2	115->116	0.65439	4.4641	277.74	0.1572	9.7934	9.9397
3	114->116	0.62854	4.9920	248.36	0.2789	-6.8888	-3.397
	115->118	0.22028					
4	109->116	0.32015	5.5407	223.77	0.0014	0.4584	0.8065
	110->116	0.36212					
	111->116	0.43682					
5	114->116	-0.2531	5.8929	210.40	0.3381	3.3154	3.7171
	114->118	0.251					
	115->118	0.58283					
6	114->118	0.60616	6.2529	198.28	0.6328	15.7433	16.853
	115->118	-0.28176					
7	108->116	0.3812	6.6249	187.15	0.0775	25.2936	25.7548
	109->116	0.27182					
	110->116	-0.33239					
8	108->116	0.48291	6.8284	181.57	0.0822	-2.3342	-3.0248
	112->116	0.22491					
	113->116	-0.34848					
9	107->116	-0.28403	7.0193	176.63	0.0365	-0.7468	-4.302
	113->116	0.36951					
	115->117	-0.34093					
10	113->116	0.3002	7.0349	176.24	0.0063	-7.9662	-7.6718
	115->117	0.52196					
11	103->116	-0.27987	7.1373	173.71	0.0294	-4.5385	-5.3278
	106->116	0.21749					
	107->116	0.38414					
	113->116	0.30001					
12	112->117	0.53743	7.2125	171.90	0.0071	0.8174	0.5312
	113->117	-0.22115					
13	115->119	0.29851	7.3910	167.75	0.0453	6.3175	6.1155
	115->120	0.35415					
	115->121	-0.25402					
14	110->117	0.21598	7.4554	166.30	0.0347	-2.5647	-1.2294
	111->117	-0.3617					
	114->117	0.42785					

15	100->116	-0.28555	7.4903	165.53	0.0106	-11.9015	-12.743
	111->116	0.33746					
	111->118	-0.24136					
16	103->116	0.37747	7.5339	164.57	0.0089	2.972	2.6266
	112->116	0.40885					
17	111->117	0.27211	7.5783	163.60	0.0236	10.3374	11.1782
	114->117	0.39209					
	115->120	0.21859					
18	113->119	0.55612	7.5975	163.19	0.0106	5.1248	1.9453
19	114->117	-0.26187	7.6194	162.72	0.0022	-7.6866	-7.94
	115->120	0.31888					
	115->121	0.21537					
	115->122	0.28963					
20	110->118	0.37155	7.7286	160.42	0.0038	-1.5491	-1.8218
	111->118	0.29128					
	112->116	0.21726					
21	114->119	0.2468	7.7943	159.07	0.0749	5.8943	5.3731
22	105->117	-0.33357	7.8134	158.68	0.0049	-2.1913	-3.9603
	106->117	0.37259					
	109->117	-0.28892					
23	109->116	0.33207	7.8317	158.31	0.0010	-3.3838	-3.2884
	112->116	0.34152					
24	98->116	0.23425	7.8540	157.86	0.0128	-29.6954	-27.9268
	111->116	0.22922					
	115->119	0.25327					
25	114->120	-0.23833	7.9210	156.53	0.0409	-21.4256	-20.2686
	114->121	0.26698					
	114->122	0.21307					
	115->119	0.24967					
26	109->118	0.31612	7.9446	156.06	0.0774	-10.2189	-12.3992
	111->118	0.34255					
27	112->119	0.43003	7.9683	155.60	0.0186	41.7807	46.6183
	113->121	0.25673					
28	115->122	-0.28629	8.0748	153.54	0.0028	1.2007	-0.2612
	115->123	0.40498					
	115->125	0.28137					
29	114->119	-0.23647	8.1062	152.95	0.0097	0.3866	0.5986
	114->124	0.29441					
	114->130	0.25104					
30	105->116	0.3313	8.1190	152.71	0.0031	-14.108	-15.0937
	106->116	0.38546					
31	108->118	-0.21535	8.1631	151.88	0.0200	10.64	9.9468
	113->118	0.35056					
	114->120	0.28624					
32	113->118	-0.28526	8.1792	151.58	0.0556	-7.305	-10.3466
	114->120	0.29656					
33	110->117	-0.22177	8.1983	151.23	0.0233	18.4959	20.6144
	114->120	0.22323					
34	107->118	0.25298	8.3034	149.32	0.0426	24.4212	25.8048
	108->118	0.38796					
	113->118	0.31514					

35	114->119	0.28645	8.3800	147.95	0.0032	-6.6088	-8.2174
	114->120	-0.25196					
	115->130	-0.21588					
36	101->117	0.26337	8.4245	147.17	0.0144	-9.6333	-5.035
	104->117	0.30845					
	113->120	0.2403					
	113->121	0.22673					

[a]Number of the excited states; [b]Only transitions with contribution over 10.0% were listed; [c]Configuration-interaction coefficient; [d]Excitation energy; [e]Wavelength; [f]Oscillator strength; [g]Rotatory strength in velocity form (10^{-40} cgs); [h]Rotatory strength in length form (10^{-40} cgs).
